# B-UMCS: Blockchain-enabled Unified Medical Consultancy Service

**DOI:** 10.1371/journal.pone.0310603

**Published:** 2024-12-02

**Authors:** Albatoul Almohana, Iman Almomani, Walid El-Shafai

**Affiliations:** 1 Security Engineering Lab, Computer Science Department, Prince Sultan University, Riyadh, Saudi Arabia; 2 Computer Science Department, King Abdullah II School of Information Technology, The University of Jordan, Amman, Jordan; 3 Department of Electronics and Electrical Communications Engineering, Faculty of Electronic Engineering, Menoufia University, Menouf, Egypt; National University of Sciences and Technology NUST, PAKISTAN

## Abstract

The advent of blockchain technology within the healthcare domain has signified a paradigm shift, transitioning from an emerging trend to an essential infrastructure component that ensures decentralization, transparency, integrity, and persistent availability. Despite its potential, the healthcare sector has not fully capitalized on the vast array of benefits blockchain technology offers. Most existing works utilized blockchain technology within a specific healthcare entity’s services but not among several healthcare organizations. They notably lack the provision for direct communication and knowledge transfer between doctors from different hospitals. Therefore, this paper introduces a pioneering Blockchain-based Unified Medical Consultancy Service (B-UMCS) that leverages blockchain’s robustness to revolutionize telehealth services by (a) alleviating the shortage of medical expertise through facilitating the interconnection of physicians from diverse hospitals and geographical areas onto a consolidated platform, (b) promoting the seamless sharing of medical consultations, electronic health records (EHRs), and expert insights while upholding rigorous security and privacy protocols, (c) integrating the inherent security mechanisms of blockchain with the distributed data storage functionality offered by the Interplanetary File System (IPFS). This work details the B-UMCS’s components, interactions, smart contracts, protocols, algorithms, storage and transmission of EHRs, and their corresponding implementations. The evaluations of the proposed B-UMCS reveal that it secures and facilitates the sharing of EHRs and enables healthcare professionals to collaborate and exchange expertise seamlessly across institutional boundaries. They are additionally ensuring that healthcare providers can offer their knowledge in an efficient and scalable manner. Overall, B-UMCS not only addresses the current challenges in healthcare data security and accessibility but also opens new avenues for collaboration and knowledge sharing among healthcare professionals, ultimately contributing to improving patient care quality.

## Introduction

The field of healthcare is crucial in maintaining the health of individuals and communities, driving numerous research and innovation efforts aimed at improving global healthcare services. These efforts focus on expanding access to these services, enhancing health outcomes [1]. Telehealth has emerged as a key technology in this advancement, utilizing telecommunications to improve healthcare delivery, support, and education [2, 3]. Recent technological integrations in healthcare have improved service quality, data accuracy, accessibility, and timeliness [4, 5]. However, these benefits also bring security and privacy risks, highlighted by increasing cyberattacks on healthcare systems aiming to steal patient data [6, 7].

To overcome security and privacy challenges in healthcare, providers are increasingly adopting secure technologies such as blockchain [8–12]. Research in this area is expanding, with applications aimed at transforming healthcare delivery, including managing appointments and facilitating secure virtual consultations and exchange of electronic health records (EHR) [13–16]. Despite advancements in telehealth and blockchain, a unified blockchain platform that enables effective collaboration among hospitals is yet to be developed. Such a platform would allow healthcare professionals from various regions and specialties to communicate and consult collectively, potentially enhancing patient care. However, the current solutions do not adequately support such wide-ranging collaboration. The necessity for this capability was particularly highlighted during the COVID-19 pandemic, as healthcare workers faced resource shortages and high demands, which a collaborative platform could help mitigate by improving communication and resource distribution across global networks [17–19].

Our proposed system in this paper represents a blockchain-based framework designed to facilitate seamless remote interactions among physicians from disparate hospitals, functioning as a virtual conduit for secure communication and collaboration among medical personnel situated in varied locales. This proposed platform is engineered to support the exchange of crucial information, solicit expert consultations, securely transfer EHR, and share insights regarding patient care strategies, all within a secure environment unhampered by geographical constraints. Aimed at optimizing the dissemination of vital data and coordinating healthcare initiatives, the system promises to significantly augment the efficacy and efficiency of medical services, safeguarding patient privacy in the process. The primary goal of this initiative is to harness blockchain technology to ensure the security, availability, and integrity of data processed by the system, thereby bolstering healthcare security. Moreover, the system endeavors to mitigate the impact of physician shortages within specific healthcare facilities, thereby enhancing the quality of care and facilitating improved patient recovery and monitoring processes, all while rigorously maintaining the security and privacy of patient data.

Thus, this work introduces a blockchain-based healthcare consultancy service (B-UMCS), developed in response to challenges highlighted by the COVID-19 pandemic. This system is designed to facilitate comprehensive consultations and medical plans, enhancing collaboration among medical professionals across geographical boundaries. Here are the key contributions of our work:

1. The B-UMCS provides a blockchain-enabled platform that leverages collective medical expertise to address the shortage of medical professionals and enhance patient care over distance.
2. Our research critically assesses how blockchain technology is integrated within healthcare, contrasting existing methods with our B-UMCS to highlight its unique advantages and areas for improvement.
3. We implement a blockchain system that facilitates its integration into healthcare practices, promoting the adoption of advanced technologies and contributing to the sector’s modernization and efficiency.
4. The system’s effectiveness can be verified in real-time scenarios and it is expected to improve healthcare delivery, patient outcomes, and ensure data security.

The structure of the remainder of this paper is organized as follows. Section 2 provides the summary of the basic concepts and the recent related studies. Section 3 describes the proposed system in detail, explaining its design, functionalities, and how it leverages Blockchain technology to facilitate secure and efficient healthcare communication and collaboration. Section 4 discusses the implementation of our system and the outcomes of its application, offering insights into its efficacy and potential impact on healthcare delivery. Finally, Section 5 concludes the paper and summarizes some future suggestions.

## Basic concepts and related work

This section provides a summarized background on Blockchain technology, outlining its key components. It also reviews related works, offering a critical analysis of prior research and developments in the field. We also compare blockchain technology with traditional Database Management Systems (DBMS) and address the scenarios presented in the recent related studies [20, 21].

### Basic concepts

**Fig 1:**
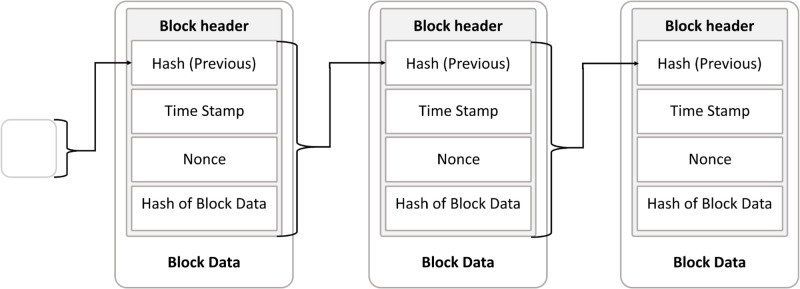
Block data structure.

This subsection discusses the foundational elements and related applications of Blockchain technology in healthcare, alongside discussing Telehealth and Electronic Health Records (EHRs). Key points include:

- **Blockchain Technology**: It is described as a series of cryptographically linked blocks, this technology provides a secure, immutable digital ledger, initially used in Bitcoin’s architecture. It operates on a decentralized Peer-to-Peer network, with transactions validated by miners through consensus mechanisms like Proof-of-Work and Proof-of-Stake, ensuring data integrity and security [22–25].
- **Block**: A block stores transaction data, with each block containing a header and body, where the header includes metadata such as block number, previous block hash, timestamp, block size, and nonce [25–27]. Fig 1 demonstrates the block details the transactions and their specific information.
- **Hash Function**: It ensures the integrity and security of data on the blockchain, featuring collision resistance, pre-image resistance, and second pre-image resistance, making it difficult to alter or reverse-engineer data [25, 28].
- **Nonce**: A crucial component in the Proof-of-Work (PoW) consensus, used to generate a unique hash for block validation [25, 28].
- **Transaction**: It represents transfers of assets such as cryptocurrencies directly between parties without intermediaries. Transactions are essential for block creation and include inputs (specifying what is transferred) and outputs (specifying the recipient) [28].
- **Telehealth**: It uses telecommunications technology to provide remote healthcare services, improving access and quality of care while facing challenges in data security and privacy [1, 29, 30].
- **Electronic Health Record**: It digitizes patient health information for easier access and management, reducing medical errors and facilitating research. However, its implementation poses financial challenges and potential disruptions in healthcare processes [31–33].

#### Blockchain technology vs. traditional DBMS

Blockchain technology and traditional DBMS differ significantly in terms of data management, security, and operational paradigms [34]. Below, we outline the key differences and highlight the necessity of blockchain in our proposed system.

**(a) Data Management**
  **(1) Traditional DBMS**:
    - Centralized architecture with a single point of control.
    - Relational data models with structured query language (SQL) for data manipulation.
    - User can create, read, update, and delete the data.
    - Vulnerable to single points of failure and data breaches.
  **(2) Blockchain Technology**:
    - Decentralized and distributed architecture with no single point of control.
    - Immutable ledger ensuring data integrity and transparency.
    - The user can read and append-only new data blocks, which helps record the history of all data without being able to temper it.
    - Enhanced security through cryptographic techniques and consensus mechanisms.
  So, traditional DBMS systems, while efficient for structured data management, are prone to single points of failure and data manipulation. In contrast, blockchain provides a tamper-proof and decentralized approach, ensuring data integrity and resilience against attacks.
**(b) Security and Privacy**
  **(1) Traditional DBMS**:
    - Relies on access control mechanisms and encryption to protect data.
    - Centralized control increases vulnerability to cyberattacks.
  **(2) Blockchain Technology**:
    - Utilizes public and private keys for secure data access.
    - Decentralization reduces the risk of single points of failure and enhances data security.

Therefore, in a blockchain-based healthcare system, patient data is encrypted and distributed across multiple nodes, making it significantly harder for unauthorized parties to compromise the data, as opposed to a centralized DBMS where breaching the central server could expose all data. For example, in [20], the authors discussed the application of blockchain technology in ensuring data integrity and transparency in a distributed system. They highlighted the use of consensus mechanisms to achieve agreement among distributed nodes. Similar to [20], our B-UMCS system exploits blockchain’s consensus mechanisms to ensure that all healthcare providers in the network have a consistent and tamper-proof view of the data. This is crucial for maintaining the integrity of EHRs across different hospitals. In addition, in [21], the authors explored how blockchain can be used to handle massive data dissemination in an Industrial Internet of Things (IIoT) environment, focusing on scalability and data security. In line with [21], our B-UMCS system addresses scalability by utilizing the IPFS for efficient data storage and retrieval. Additionally, our use of public key infrastructure (PKI) and role-based access control (RBAC) ensures that only authorized personnel can access sensitive patient data, thus maintaining data security and privacy.

For further clarification, the integration of blockchain technology in B-UMCS involves several key components and processes:

**1. Blockchain Network**: The Ethereum blockchain is the backbone of the B-UMCS, providing a secure and immutable ledger for recording transactions. Each transaction, such as a consultation request or the sharing of an EHR, is recorded on the blockchain, ensuring transparency and traceability.
**2. Smart Contracts**: Smart contracts are deployed on the Ethereum blockchain to manage interactions between healthcare providers. These contracts define the rules and conditions for various operations, such as initiating consultations, sharing EHRs, and updating patient records. Smart contracts ensure that these operations are executed automatically and transparently.
**3. Decentralized Storage with IPFS**: EHRs and other large datasets are stored off-chain using the IPFS. This decentralized storage solution ensures that data is distributed across multiple nodes, enhancing accessibility and resilience. The blockchain stores cryptographic hashes of the records to ensure data integrity and verify authenticity.
**4. Secure Data Access**: Data security is maintained through advanced encryption techniques. PKI is used to control access to sensitive information, ensuring that only authorized healthcare providers can access patient records. RBAC is implemented to further restrict access based on user roles.

### Literature review

In this section, we provide a comprehensive review of the existing literature on blockchain technology in healthcare, focusing on its applications, benefits, and limitations. The systematic approach includes defining the scope, selection criteria, and summarizing the findings of relevant studies.

This systematic literature review provides a comprehensive overview of the current state of blockchain applications in healthcare. It highlights the advancements, challenges, and future directions for research in this field. By addressing the gaps identified in existing studies, the proposed B-UMCS aims to enhance the security, efficiency, and collaboration among healthcare providers, ultimately improving patient care.

Therefore, this subsection reviews literature on telehealth systems, highlighting their crucial role in modern healthcare and noting a common shortfall: many systems do not provide a fully comprehensive telehealth solution. Research is now increasingly focused on enhancing the secure sharing and access of EHR using blockchain technology, cryptographic methods, and access control mechanisms.

#### Scope and selection criteria

The scope of this literature review includes studies and articles published in peer-reviewed journals, conferences, and reputable sources in the last recent years from 2018 to 2024. The selection criteria for including studies in this review are:

1. The relevance to blockchain technology applications and consultancy services in healthcare.
2. The focus on EHRs, telehealth services, and data security.
3. The studies that provide empirical data, case studies, or theoretical frameworks.

#### Search strategy

A systematic search was conducted in several academic databases, including PubMed, IEEE Explore, ACM Digital Library, ScienceDirect, and Google Scholar. Keywords used in the search included “blockchain in healthcare,” “medical consultancy service,” “electronic health records,” “telehealth,” “healthcare consultation services,” “data security in healthcare,” and “blockchain applications in medicine.” The search was refined using Boolean operators and filters to identify the most relevant studies.

#### Summary of findings

The following paragraphs summarize the key findings from the current studies, categorized into different themes in terms of (a) Blockchain for EHRs, (b) Blockchain for Telehealth Services, and (c) Security and Privacy in Blockchain Healthcare Systems.

The study by Xiao et al. discussed a blockchain system specifically for EHRs using consortium blockchain technology, where each node holds a ledger copy with EHRs and governed permissions [35]. Sheela et al. also explored a blockchain approach for EHR management, which while innovative, faced challenges like increased storage costs and slower network distribution [36]. Furthermore, while these frameworks facilitated communication among multiple hospitals, they notably lack the provision for direct communication and knowledge transfer between doctors from different hospitals. This limitation underscores a critical gap in the existing telehealth infrastructure, highlighting the need for a more integrated system that not only secures and facilitates the sharing of EHRs but also enables healthcare professionals to collaborate and exchange expertise seamlessly across institutional boundaries.

In their research, Praveen et al. proposed a novel blockchain-based system for managing EHRs, which incorporated off-chain storage solutions to maintain system efficiency and address the challenges of on-chain data storage. This system aimed to enhance communication among healthcare stakeholders including patients, doctors, and insurance providers [37]. Additionally, Hassija et al. introduced a patient-centered blockchain framework using the Ethereum blockchain, designed to recommend specialists for minor medical consultations. This system allowed patients to log in, describe their medical conditions, and interact directly with doctors, thus improving patient engagement and aiming to protect privacy, although the specific privacy protection methods are not detailed in their study [38].

In their study, ANOOHYA et al. [39] introduced a blockchain-based system utilizing the InterPlanetary File System (IPFS) for storing digital content, which enhances privacy, non-repudiation, and immutability of actions within the system. This system facilitated various medical interactions but lacked direct doctor-to-doctor interactions. Kordestani et al. [40] developed HapiChain, a blockchain-based monitoring system with a three-layer architecture designed to enhance functionality and security. Puneeth et al. [41] proposed a blockchain framework that improves the privacy and security of EHRs by using IPFS and hybrid encryption techniques, optimizing the search mechanism and reducing time and space complexity. This system allows patients to control their EHRs’ visibility to doctors, but limits doctors to viewing EHRs without enabling them to diagnose or create treatment plans. Each of these systems contributed uniquely to the blockchain and healthcare domain, showcasing the potential of blockchain technology in enhancing the security, privacy, and efficiency of healthcare services. However, they also highlighted the need for further development, particularly in enabling more interactive and collaborative functionalities for healthcare professionals, and in providing comprehensive details on security measures and system operations.

In the works of Cerchione et al. [42] and Uddin et al. [43], the authors explored blockchain-based networks designed to enhance the quality of healthcare services by generating valuable data sources for science and research. These studies presented effective implementations of telehealth services with a strong focus on health data protection. However, they did not address the primary goal of our research, which is to mitigate resource shortages within hospitals by facilitating more efficient resource allocation and utilization. Hassan et al. [44] introduced a private blockchain solution to address healthcare challenges by enforcing restrictions and assigning access based on participant roles, utilizing Ethereum for activity tracking and IPFS for data storage. This system enhanced telehealth security and efficiency but had limitations in simulating real-world operational dynamics due to its local Ethereum blockchain implementation and lacked a decentralized application for end-user interaction. Bawany et al. [3] developed BlockHeal, a comprehensive telehealth framework that integrates all essential healthcare services onto a single platform. This framework, which utilizes blockchain technology and Hyperledger fabric, aims to include all stakeholders (patients, doctors, health ministries, pharmacists, and insurers) within a secure, reliable, and timely healthcare system, facilitated by a suite of decentralized applications. Despite its complexity and ambition to streamline healthcare service delivery, the framework did not facilitate doctor-to-doctor communication for case management, and details on the consensus mechanisms and algorithms used in the system’s implementation are not provided. These varied approaches to utilizing blockchain technology in healthcare illustrate the potential for enhancing the security, efficiency, and accessibility of healthcare services. However, they also highlight the need for further development to address specific challenges such as facilitating professional collaboration and providing detailed technical specifications for system implementation.

Lee et al. [45] developed a blockchain-based infrastructure designed to establish a global health record exchange platform that ensures the confidentiality, integrity, and availability of health records for efficient health management. This platform used Health Level 7 Fast Healthcare Interoperability Resource (HL7 FHIR) standards to format data, enabling the international exchange of Personal Health Records (PHRs) across healthcare institutions and between patients and doctors. The system architecture included a PHR management interface for user engagement and a blockchain exchange system constructed on Ethereum, which utilized a private blockchain network with a proof of authority consensus model for transaction validation. However, the specific database used for storing EHRs was not detailed, including whether it was cloud-based or not, and the security measures applied during the sharing of EHRs were limited to blockchain constraints, without additional controls. Christodoulou et al. [46] introduced a decentralized, blockchain-based framework aimed at enhancing privacy in health information exchanges and empowering patients to manage their medical data and control access permissions, thus maintaining their autonomy. Additionally, research by Patil et al. [47] developed a framework for distributing EHRs that combines blockchain technology with the decentralized IPFS within a cloud environment. This framework proved effective in securely transferring data and protecting sensitive patient information, offering significant improvements over traditional data sharing models, such as enhanced access control, reduced network latency, and heightened security and privacy. However, this system primarily facilitated patient actions in uploading and sharing EHRs, without detailing the involvement or functionalities available to healthcare professionals, particularly doctors.

#### Critical analysis of literature studies and their gaps

The review of existing literature, summarized in Table 1, highlights key challenges in ensuring privacy and security of EHRs and in facilitating secure information exchange within peer networks. It is observed that the introduction of blockchain technology into healthcare has brought significant advancements, particularly in enhancing data security, transparency, and interoperability. Furthermore, Scholarly work shows a strong preference for patient-centric models, which significantly improve patient autonomy and security in managing health data. However, these models typically overlook the critical need to address real-time challenges in healthcare, such as the scarcity of healthcare providers in various settings. However, several gaps remain in the current literature, which our study aims to address through the proposed B-UMCS.

**Table 1:** Summary of the comparison between our work and the related studies.

Ref.	Description	Security Features	Data Storage	Blockchain Role	Blockchain Type	Doctor to Doctor Consultation
[35]	Proposed a blockchain-based system to enable the distribution of EHRs among participants	Privacy	On-chain	Data storage	Ethereum	No
[36]	Proposed the integration be tween EMR and EHR on blockchain for more reliability	Confidentiality	Or-chain	Data storage	Hyperle-dger Fabric	No
[37]	Developed a blockchain based system for Secure EHR management	Confidentiality	Off-chain	Tracking activities	Private Ethereum	No
[38]	Proposed a blockchain based system for medical consultation between patient and healthcare providers	Privacy	Or-chain	Data storage	Ethereum	No
[39]	Developed a blockchain based system to which enables the sharing of EHR and conduct telehealth services	Integrity	Off-chain	l Tracking activities	Ethereum	No
[40]	Developed a blockchain based system for enabling patients diagnose conditions through remote consultations	Confidentiality	Off-chain	Tracking activities	Ethereum	No
[41]	Proposed a blockchain-based system for patient-centred telehealth services	Confidentiality	Off-chain	Tracking activities	Ethereum	No
[42]	Developed a permissioned blockchain for transferring EHRs	Integrity and Privacy	Off-chain	Tracking activities	Ethereum	No
[43]	Proposed a blockchain architectures are utilized for EHRs to establish repository system	Privacy	On-chain	Data storage	Hyperledger Fabric	No
[44]	Developed a blockchain-based solution to demonstrate how specifically three important telehealth services: namely, teleconsultation, drug administration, and medical testing	Integrity, Im mutability, and Accountability	Off-chain	Tracking activities	Ethereum	No
[3]	Developed a blockchain-based system to integrate all health care services	Privacy	Or-chain	Data storage	Hyperle-dger Fabric	No
[45]	Developed a blockchain-based platform to ensure secure transaction of EHRs	Confidentiality, Integrity, and Availability	On-chain	Data storage	Ethereum	No
[46]	Developed a blockchain-based framework for health information exchange	Confidentiality and Integrity	Off-chain	Tracking activities	Ethereum	No
[47]	Developed a decentralized blockchain-based system for sharing Heath Data	authenticity and integrity	Off-chain	Tracking activities	Ethereum	No
Ours	B-HMCS: Blockchain-based Healthcare Consultancy Service	Confidentiality, Integrity, and Availability	Off-chain	Tracking activities	Ethereum	Yes

Therefore, despite the promising applications of blockchain in healthcare and medical consultancy services, the following shortcomings have been identified in the existing literature:

**1. Limited Inter-Hospital Collaboration**: Current blockchain-based healthcare systems primarily focus on intra-hospital applications and lack mechanisms for effective inter-hospital collaboration. They also do not adequately support seamless communication and knowledge transfer among healthcare providers and practitioners across different institutions. Improving patient outcomes and sharing professional expertise requires an integrated system for seamless doctor-to-doctor contact.
**2. Scalability and Data Management**: Many studies highlight the scalability issues associated with blockchain technology, particularly in managing large volumes of healthcare data. Traditional blockchain implementations often struggle with data storage and retrieval efficiency, which is critical for healthcare applications involving extensive EHRs.
**3. Security During Data Transfer**: Many systems rely on blockchain’s inherent features, like immutability and decentralization, to secure EHRs. However, these systems often lack additional security measures during data transfer; they ensure data security at rest. Consequentially, they overlook the vulnerabilities associated with data transmission, making the data susceptible to breaches during transit.
**4. Real-Time Applicability**: There is a lack of real-time applicability in existing blockchain-based healthcare solutions. Most studies focus on theoretical frameworks and simulations without demonstrating practical implementations that can operate efficiently in real-world healthcare settings.
**5. Integration with Emerging Technologies**: Limited research has been conducted on integrating blockchain with other emerging technologies such as artificial intelligence (AI) and the Internet of Things (IoT) to enhance healthcare services. This integration could potentially improve diagnostic accuracy, patient monitoring, and overall healthcare delivery.

#### How B-UMCS addresses the gaps in the literature

The proposed B-UMCS aims to address several critical gaps in the current literature on blockchain applications in healthcare through the following features and improvements:

**1. Enhanced Inter-Hospital Collaboration**: B-UMCS facilitates seamless communication and knowledge transfer among healthcare providers and practitioners from different hospitals and regions. By leveraging smart contracts and a decentralized architecture, B-UMCS enables real-time consultations and collaborative decision-making, thereby addressing the limitations of current systems.
**2. Efficient Data Management with IPFS**: B-UMCS integrates the IPFS for off-chain and decentralized data storage to tackle scalability and data management challenges. This approach ensures efficient storage and quick retrieval of large volumes of healthcare data, enhancing the system’s scalability.
**3. Secure Data Transfer Mechanisms**: B-UMCS employs advanced encryption techniques and PKI to secure data during transfer. By implementing RBAC and end-to-end encryption, the system ensures that data remains protected throughout the transmission process.
**4. Real-Time Implementation**: Our study demonstrates the real-time applicability of B-UMCS through practical implementation and evaluation in healthcare settings, showcasing its capability to operate efficiently and effectively in practice.

Consequently, in response to these findings, our paper introduces a secure B-UMCS system designed for medical consultancy services. This system aims to enhance the security and privacy of EHRs and facilitate robust information exchange among healthcare providers, potentially easing provider shortages and enhancing the quality of healthcare services offered. The next section details the proposed system’s architecture, functionalities, and approach to addressing the limitations identified in existing research.

## B-UMCS: Blockchain-enabled Unified Medical Consultancy Service

**Fig 2:**
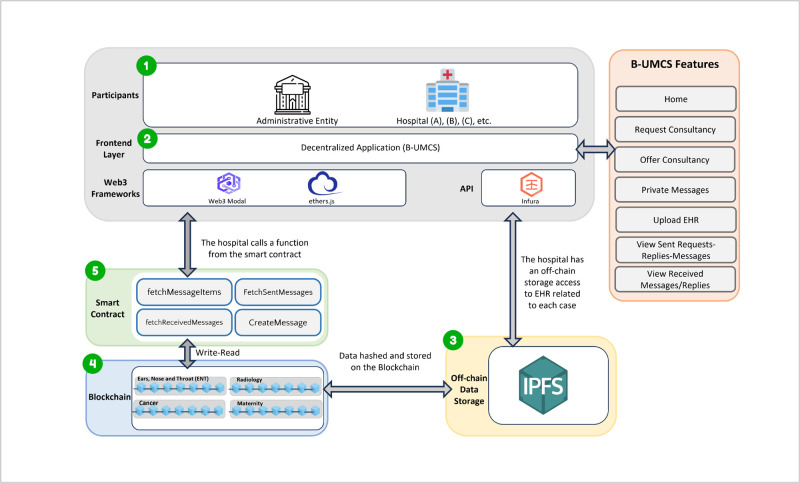
Proposed system.

**Fig 3:**
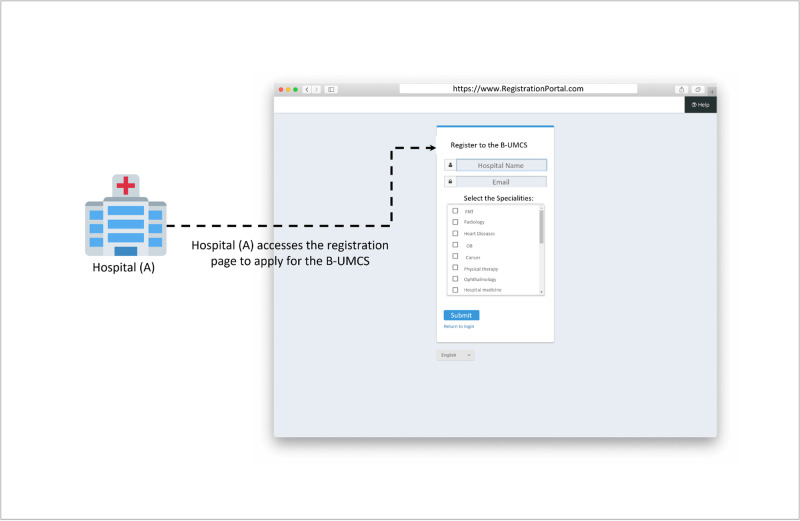
Hospital (A) applies for the B-UMCS.

**Fig 4:**
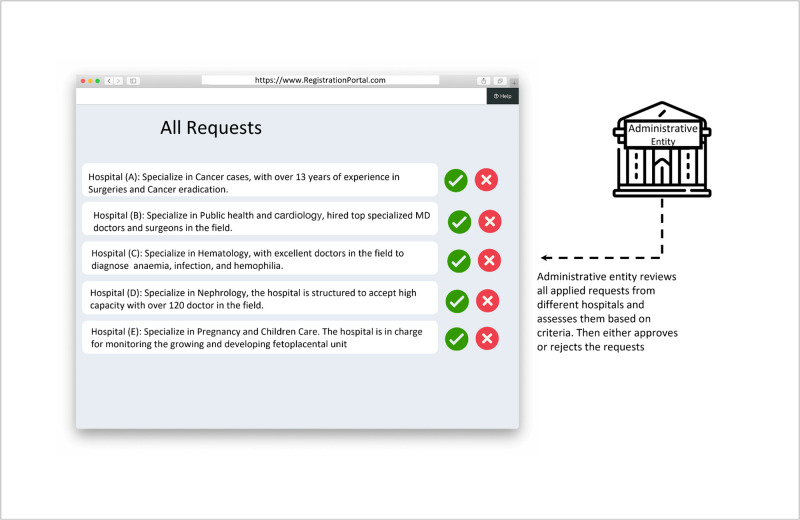
MOH reviews all hospital requests.

**Fig 5:**
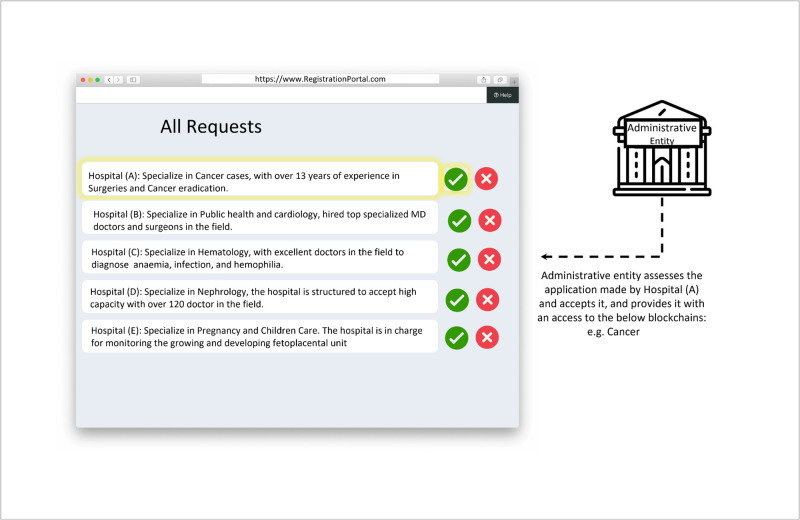
MOH approves the application of Hospital (A) on the cancer BC.

**Fig 6:**
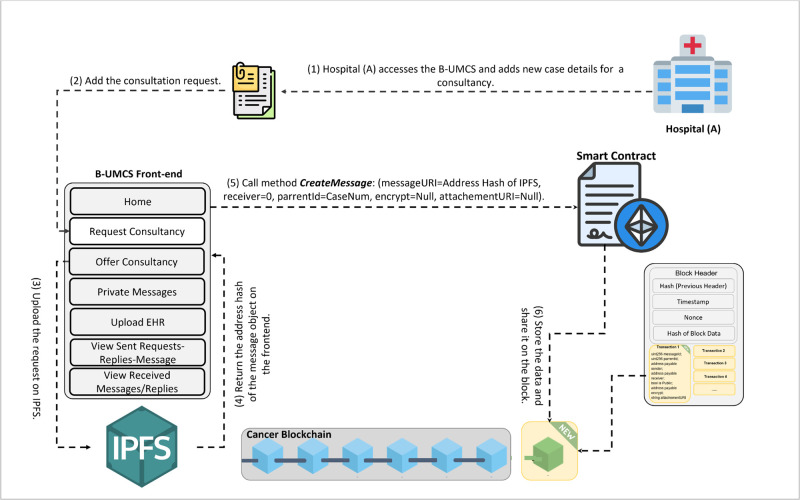
Hospital (A) requests a consultation.

**Fig 7:**
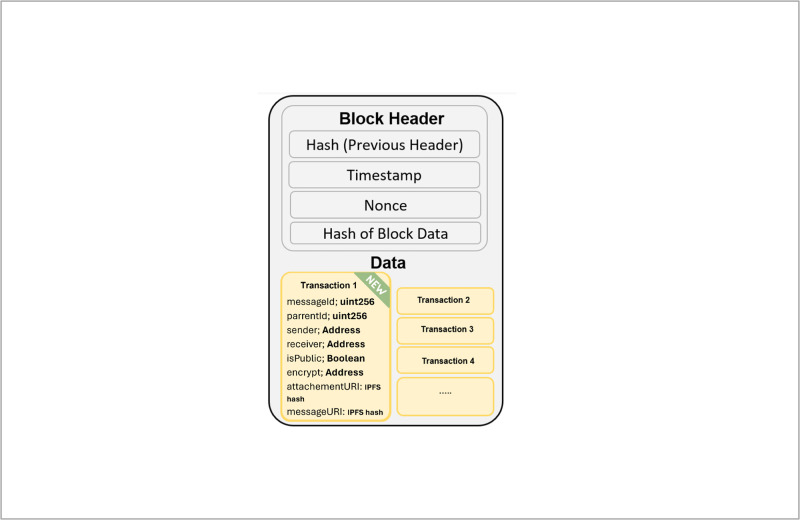
Block components.

**Fig 8:**
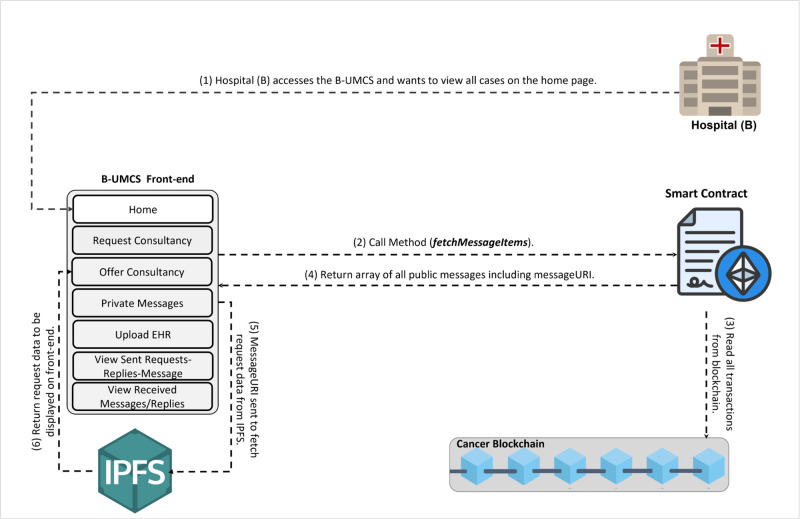
Updating the home page for all peers.

**Fig 9:**
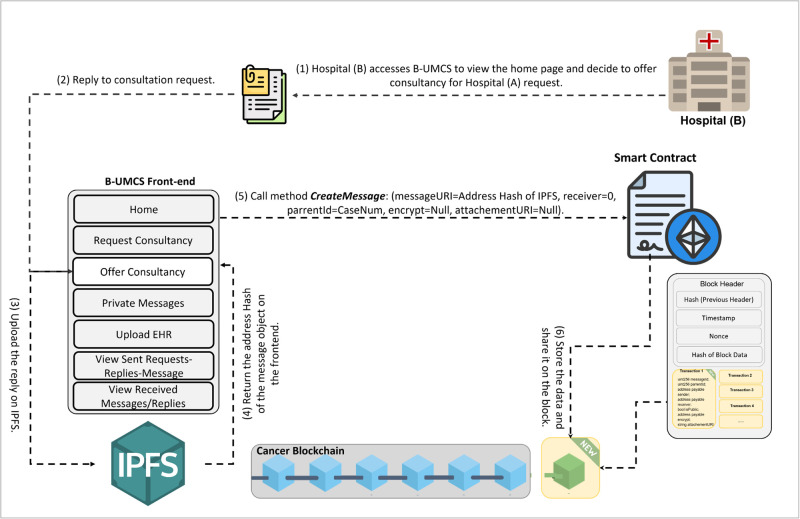
Hospital (B) replys to a request.

**Fig 10:**
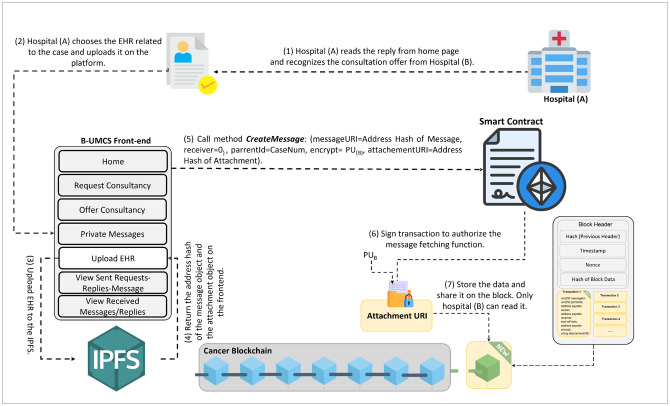
Uploading the EHR.

**Fig 11:**
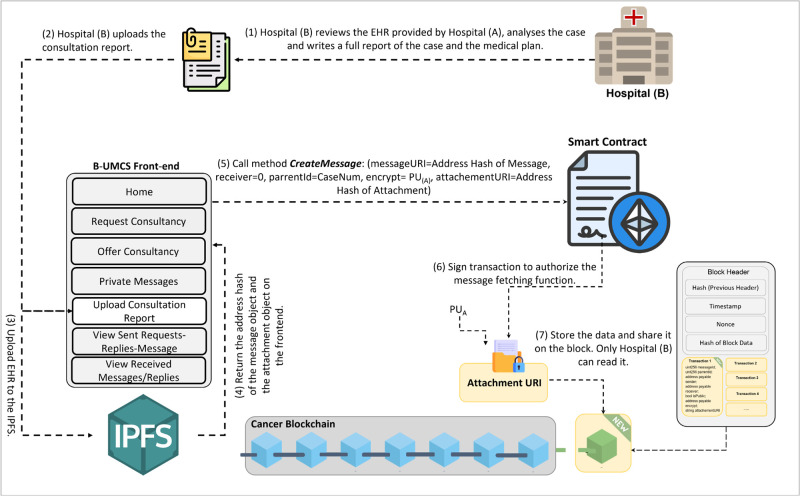
Providing the CR.

**Fig 12:**
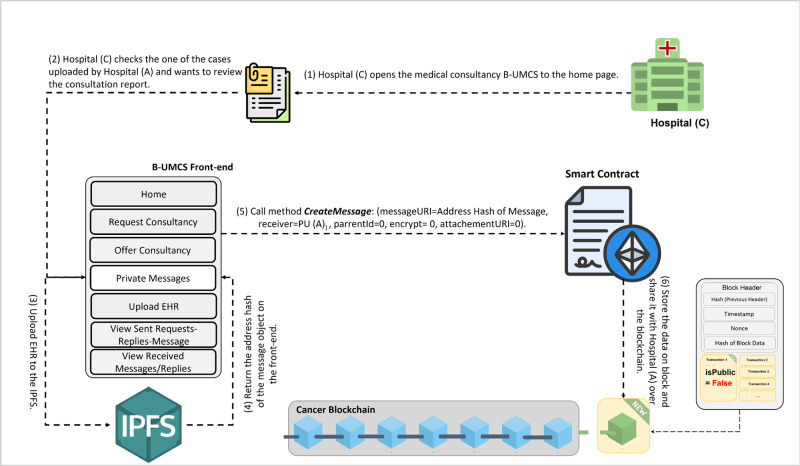
Hospital (C) sends a private message to Hospital (A).

**Fig 13:**
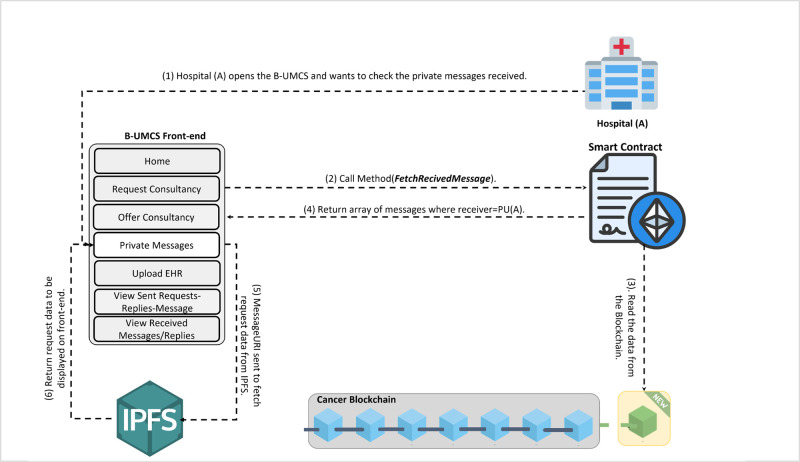
Hospital (A) receives a private message from Hospital (C).

**Fig 14:**
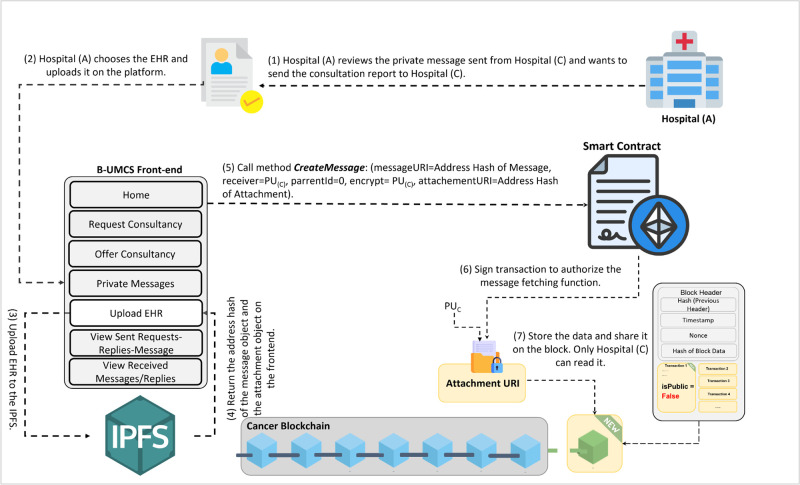
Hospital (A) sends the CR to Hospital (C).

**Fig 15:**
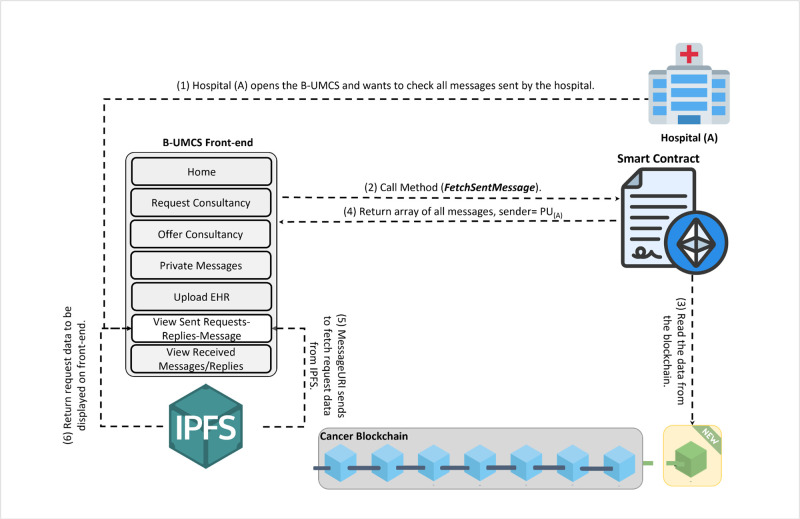
Hospital (A) checks all its sent messages.

The proposed system represents a novel approach to addressing the complexities and challenges inherent in the healthcare sector, particularly in the context of healthcare consultancy services. By leveraging blockchain technology and Non-Fungible Tokens (NFTs) which is a digital token that is maintained on a blockchain which represent ownership of digital assets, and it is used to represent ownership or proof of authenticity of a unique content or files such as EHRs [48]. The system aims to provide a secure, dependable, and innovative solution for the interaction and collaboration among doctors across various healthcare institutions. This section outlines the architecture, key components, and operational dynamics of the proposed system, explaining how it facilitates secure interactions and transactions through smart contracts, as illustrated in sequence diagrams (See Figs 2–15).

The cornerstone of the proposed system is its use of blockchain technology, which serves as a foundational layer for creating a secure and immutable record of transactions and interactions. This blockchain framework ensures that every action taken within the system, from consultation requests to the exchange of EHRs, is recorded in a tamper-proof manner. The adoption of NFTs within this ecosystem introduces a unique mechanism for representing ownership and access rights to specific healthcare data and consultations, thereby enhancing the security and specificity of data access and transfer.

Furthermore, the system incorporates the IPFS for decentralized storage, addressing one of the critical challenges in healthcare data management: the secure and efficient storage of large volumes of sensitive data. By utilizing IPFS, the system ensures that critical documents and EHRs are stored in a manner that is both secure against unauthorized access and resilient against data loss.

The operational dynamics of the proposed system are defined by a series of smart contracts deployed on the blockchain. These smart contracts facilitate various stages of the healthcare consultancy process, including:

1. **Consultation Request and Acceptance**: Doctors or healthcare providers can request consultations for their patients by creating a new transaction on the blockchain. This transaction, represented by an NFT, specifies the details of the consultation request and the EHRs to be reviewed.
2. **Secure Exchange of EHRs**: Upon acceptance of a consultation request, the relevant EHRs are securely exchanged via IPFS, with access rights managed through NFTs. This ensures that only authorized parties can access the sensitive information.
3. **Consultation Process**: Throughout the consultation process, all communications, recommendations, and notes are recorded on the blockchain, ensuring an immutable record of the consultancy provided.
4. **Completion and Feedback**: Upon completion of the consultation, the system facilitates the provision of feedback and any further recommendations, all of which are securely documented on the blockchain.

The presented sequence diagrams in Figs 2–15 provide a detailed visual representation of the interactions between doctors, the blockchain, and the IPFS, illustrating the step-by-step process of requesting, conducting, and completing consultations. These diagrams serve as a guide for understanding the flow of transactions and data within the system, highlighting the role of smart contracts in automating and securing these processes. To sum up, by integrating blockchain technology, NFTs, and IPFS, the proposed system aims to revolutionize the way healthcare consultancy services are provided, ensuring that healthcare providers can offer their expertise in a manner that is secure, efficient, and scalable. This system not only addresses the current challenges in healthcare data security and accessibility but also opens new avenues for collaboration and knowledge sharing among healthcare professionals, ultimately contributing to the improvement of patient care quality.

### System components

The system architecture depicted in Fig 2 illustrates a comprehensive structure for healthcare consultancy service chain. This architecture is designed to facilitate remote connections between hospitals in various locations and comprises five fundamental components: (1) the Hospital, (2) a DApp, (3) off-chain storage, (4) a blockchain infrastructure, and (5) smart contracts. Each element plays a crucial role in the system’s overall functionality:

- **Ethereum (ETH) Blockchain**: Serving as the backbone of the system, Ethereum’s blockchain is utilized for its robust smart contract capabilities. These smart contracts are essential for automating processes and ensuring transparent and tamper-proof interactions on the blockchain. Ethereum has been a game-changer in the field of blockchain technology since its inception in 2015 by Vitalik Buterin. It revolutionized the space by introducing the ability to execute smart contracts on the blockchain, significantly diverging from Bitcoin’s original design as a digital currency. Ethereum provides an environment through the Ethereum Virtual Machine (EVM) where developers can write and deploy custom code using Solidity, a programming language tailored for the EVM, thus enabling a myriad of applications beyond simple transactions [49, 50].
- **Interplanetary file system (IPFS)**: For distributed storage, the system employs IPFS to manage and store the metadata associated with NFTs, which in our project, the text of the consultation requests an, EHRs, and CRs. This is done by enabling the DApp to captures the inputs from the hospital and upload it on the IPFS to store it on IPFS and create a unique hash to be able to trace the data or file from IPFS. This ensures the integrity and uniqueness of files across a global namespace, with each file indexed and retrievable based on its cryptographic hash. Thus, the implementation of IPFS as a distributed storage system is critical for handling the metadata associated with NFTs. By employing content addressing and a peer-to-peer hypermedia protocol, IPFS ensures that each file is unique within a global namespace through content identifiers (CIDs). This allows for efficient indexing and retrieval of files based on their cryptographic hash, making it easier for hospitals to access and share files securely and reliably [51].
- **Smart contract**: Acting as the automated enforcers of the system’s rules, smart contracts allow for secure and consistent execution of agreements, with the ERC-721 standard providing a unique identity for every NFT within the system [52]. So, smart contracts on the blockchain act as autonomous, self-executing contracts with the terms directly written into code. They enable transparent and secure interactions with the blockchain ledger. Utilizing the ERC-721 standard for NFTs, the smart contracts in the proposed system assign a distinct identity to each NFT, allowing for the tracking of transactions and ownership history. The system includes key smart contract functions such as *CreateMessage, FetchSendMessages, FetchReceivedMessages, and FetchMessageItem*, which collectively manage the communication and transaction processes within the system
- **Hospitals**: In this system, hospitals are the primary actors and are represented as individual users on the blockchain. Each hospital can initiate the creation of NFTs, which symbolizes their role in starting consultations and sharing EHRs. The blockchain’s transparent nature allows for the traceability of all actions back to the initiating hospital, ensuring accountability and provenance.
- **Decentralized Application**: The DApp provides an interface for hospitals to interact with the smart contracts, manage NFT communications, and access the distributed storage resources, facilitating a user-friendly environment for the exchange of healthcare information. This interaction facilitates the management and exchange of NFTs, allowing hospitals to perform various functions related to medical consultancy services directly from the DApp. By leveraging the benefits of decentralization, the DApp ensures that the handling and distribution of NFT resources are adaptable and user-friendly.

So, the proposed system aims to create a secure, efficient, and user-centric platform for healthcare services, utilizing cutting-edge blockchain and IPFS technologies to enhance the exchange and management of health information. Through this integration, the system addresses critical issues in healthcare data security and accessibility, paving the way for improved collaboration among healthcare providers and better patient care outcomes and to achieve patient outcomes and streamlining healthcare delivery across different geographic areas.

### System interaction

The system interaction design of the proposed platform is meticulously architected to enable hospitals to securely manage and exchange EHRs, consultations, and messages, with provisions for both private and public communication channels. The platform leverages the robust cryptographic capabilities that are embedded within the blockchain technology, such as public key cryptography which is the foundational technology that the blockchain relays on it securing the transactions, digital signatures that enable secure and efficient meant to verify the transactions without decentralization needs, and cryptographic hash functions for For preserving the integrity and security of data. Employing these features is crucial to ensure the confidentiality and integrity of the data within the proposed system.

Central to the platform’s security strategy is the restricting the access of sensitive details, particularly EHRs. These records are obscured from public view and can only be accessed by the consulting doctors, ensuring patient privacy and data protection. This mechanism is vital for maintaining the confidentiality of patient information, a fundamental requirement in healthcare information systems.

The platform facilitates a range of interactions amongst doctors, including:

- **Consultancy Requests**: Doctors can initiate consultancy requests on the platform, seeking expertise from other healthcare professionals.
- **Offering Consultations**: Specialists can offer their insights on existing cases, contributing to collaborative case management.
- **Secure EHR Sharing**: The platform provides a secure means to share EHRs, ensuring that sensitive patient data is exchanged without compromising privacy.
- **Private Messaging**: Hospitals can engage in private messaging through the platform, enhancing communication between different healthcare institutions.

The system interaction also involves the Administrative entity, such as Ministry of Health (MOH), which serves as an initial governing entity. Hospitals that wish to participate in the B-UMCS must apply for access through the MOH. The MOH’s role includes the review, assessment, and approval of hospital applications to join the specialized blockchain network designated for each medical specialty. This ensures that only verified and authorized entities are granted access to the system, further reinforcing its security and integrity.

#### Hospital registration process

This section covers the three steps interaction process detailed in Figs 3–5, which illustrate the sequential steps involved in hospital applications, administrative entity’s assessments, and the subsequent approval process. These figures provide only an example representations of the workflows, delineating the various stages of interaction from application to active participation in the B-UMCS. By integrating these components, the proposed platform aims to streamline the process of healthcare consultancy, foster a collaborative environment among medical professionals, and significantly enhance the security and privacy of patient data within the healthcare system.

The process illustrated in Fig 3 depicts a streamlined approach for hospitals to register and participate in a specialized blockchain network for B-UMCS. The procedural steps are as follows:

- **Accessing a dedicated registration portal**: Hospital (A) start the registration process by accessing the registration portal.
- **Providing registration information**: Upon accessing the portal, Hospital (A) is presented with a registration interface where it is required to input critical identifiers such as its name and email address.
- **Prompting the areas of interest**: The registration form also prompts Hospital (A) to select its areas of medical specialty, for example, hospital (A) is identifying Cancer treatment as its area of interest for the B-UMCS.

As depicted in Fig 3, the registration establishes a framework for a mutual exchange of medical knowledge and services, creating a network of collaborative support that enriches the capabilities of each participating institution.

By completing this registration, Hospital (A) takes the first step toward joining a community of medical professionals dedicated to improving patient outcomes through collective expertise. The system is designed to facilitate a secure, reciprocal sharing of knowledge, wherein each hospital contributes to and benefits from the shared pool of medical specialization, thereby enhancing the quality and breadth of healthcare services available.

The process depicted in Fig 4 showcases the review phase undertaken by the MOH. It illustrates a user-friendly interface for the MOH to manage applications efficiently, highlighting the importance of a robust and transparent review system in the healthcare consultancy blockchain network. This system interaction phase is essential for maintaining the quality and reliability of the healthcare services provided through the B-UMCS.

In the review phase, the MOH evaluates applications from various hospitals seeking to join the B-UMCS. This step is crucial as it ensures that only qualified and credible institutions are allowed access to the system, thereby maintaining high standards of healthcare consultancy services. Therefore, upon receiving applications through the registration portal, the MOH conducts a thorough assessment of each hospital. This assessment is based on established criteria, likely encompassing factors such as medical expertise, years of experience, and the hospital’s technological readiness to integrate with the blockchain system. Hospitals are listed with their respective areas of specialization, such as ENT, surgeries, and cancer, alongside their years of experience in these fields. The MOH uses this information to make informed decisions on which hospitals will be granted access to the network. Each application is accompanied by two actionable options represented by check (approve) and cross (reject) icons, allowing MOH officials to easily approve or reject applications with a simple click. This streamlined process aids in expediting the review phase while ensuring accuracy and fairness in the approval process.

The transparency of the approval process is critical, and the blockchain network facilitates this by providing an immutable ledger of all actions taken by the administrative entity, ensuring that transactions are recorded in cryptographically linked blocks and broadcast over a network, guaranteeing that each entry is permanent and tamper-proof. This builds trust among participating hospitals and stakeholders. By employing such a detailed review system, the MOH acts as a administrative entity, upholding the integrity of the medical consultancy services provided through the blockchain.

The approval process as shown in Fig 5 involves the MOH reviewing and assessing the applications of various hospitals, including Hospital (A), that have applied to be part of a specialized blockchain network. The MOH’s role is critical in vetting the hospitals based on their expertise, areas of specialization, and experience to ensure the highest quality of collaborative healthcare consultation services within the blockchain network.

Upon a successful review, Hospital (A)’s application to join the blockchain network specializing in cancer treatment is accepted. The MOH grants Hospital (A) access to the network, enabling it to become a contributing member to this exclusive pool of healthcare expertise. As a result, Hospital (A) gains the capability to request and provide specialized consultations, share EHRs securely, and engage in private messaging with other network hospitals to collaborate on patient cases. This process is essential to form a secure, decentralized network of healthcare providers that leverages the strengths of blockchain technology to enhance the quality of care provided to patients. The approval and access granted by the MOH allow for a secure and private exchange of medical knowledge, fostering a community of medical practice aimed at leveraging collective expertise for improved patient outcomes.

Fig 6 delineates the essential functionality of the system’s Request Consultancy page. This interface empowers hospitals to draft and disseminate consultancy requests to peers within the network and to respond to existing inquiries. The process goes as follows

- **Drafting the Consultation request**: Hospital (A) start by navigating through the “request consultancy” page where the request can be drafted
- **Broadcasting the request**: After drafting the request, hospital (A) is ready to share the request with all peers within the blockchain. this is done through sharing the request message that sent from the DApp front-end to the IPFS.
- **Hashing the request and calling Smart Contract**: The request data is saved on IPFS, is hashed, creating a cryptographic representation of the content. This hash is then returned to the DApp front-end, and smart contract function ‘CreateMessage’ is then called.
- **Completing the Blockchain Transaction**: The response text is digitally signed to guarantee its authenticity and prevent denial of its origin. This signing uses the the Elliptic Curve Digital Signature Algorithm (ECDSA), an effective method for generating verifiable digital signatures.using the private key of the sender. This signature guarantees that the message’s origin is legitimate and that the content has not been altered, as it can be verified by anyone with access to the corresponding public key
- **Incorporating into the Blockchain and Distributing**: The signed response is incorporated into a new block on the blockchain, where it is timestamped and linked to the existing sequence, ensuring the data remains continuous and unalterable. After this block is formed, it is transmitted to all peers in the blockchain network, thereby informing all members about the current consultation and the latest information from Hospital (A).

Fig 7 illustrates the components of the block specifically the ‘Data’ section in the Fig 1. it shows the transaction’s data that has been stored on the block. The data includes (8) elements which are:

- **messageId variable**: A variable that store the value of the message identifier. This variable is automatically generated every time the ‘CreateMessage’ function 2 is invoked. This variable’s value is unique that can identify the request on the blockchain.
- **parentId variable**: this variable hold the value of the original request of the thread, indicating that this is an initial, standalone request. this variable is automatically filled every time the button ‘reply’ is clicked to reply an original request.
- **Sender Variable**: This variable holds the value of the sender’s address or public key. In Fig 21, the public key can be seen at the very end of the request message which is under the hospital name.
- **Receiver Variable**: This variable holds the value of the reciver, this variable’s value is ‘0’ whenever the request is sent to all peers on the blockchain, but hols an actual public address value when it is privately sent to one peer. Also, this variable is mainly used when the function 6 since this function checks all requests that hold the current hospital address as value in the receiver variable.
- **isPublic variable**: is boolean variable that hold either true or false value to check the request/reply status if it is public or Private. This is done through the function 2. the receiver value is checked, if it is equal to ‘0’ then the variable is assigned with true value, if not, then assigned with false. meaning this request/reply is not going to be publicly shown to all peers on the blockchain.
- **encrypt variable**: this variable holds the value of the key of the receiver that is authorized to access the EHR or the CR. This variable is used through the function 4 to restrict the access of the file hash. To ensure it is only accessible to that specific user.
- **attachementURI variable**: this variable holds the attachment’s hash that is returned from the IPFS.
- **messageURI variable**: This variable holds the value of the request/reply message’s hash that is returned from the IPFS.

Hospital (B), along with other network participants, is notified of this new case via updates on their respective home pages within the platform. This process ensures a transparent, secure, and efficient method for requesting and offering medical consultancy services (See Fig 8). This process include the following:

- **Access Home Page**: Hospital (B) access the B-UMCS DApp, and access the default home page, it refreshes to capture all public requests/replies.
- **Fetch function invoking**: ones the home pages is accessed, it invoke the function ‘FetchMessageItem’ 3, to return all messages that are (isPublic==true).
- **return array of MessageURI**: after calling the function ‘FetchMessageItem’ 3, it return an array of public MessageURI, that is then sent to the IPFS to return the actual request/replies.

Fig 9 encapsulates the process wherein Hospital (B) demonstrates an intent to engage with a case posted by Hospital (A), seeking additional information and the EHR of the patient. So, after perusing the available cases on the platform, Hospital (B) signals its willingness to delve deeper into a specific case presented by Hospital (A). To facilitate this, Hospital (B) initiates a request to access the patient’s EHR. The procedural steps are as follows:

- **Initiating a Response to Consultation Request**: Hospital (B) navigates to the ‘Reply’ section of the Request Consultancy page on the Unified BC Portal for Medical Consultancy and prepares a response to Hospital (A)’s consultation request.
- **Storing the Response**: The composed response is securely stored on the IPFS, leveraging its decentralized nature for enhanced security and integrity of data.
- **Hashing and Smart Contract Invocation**: The response data, now on IPFS, is hashed, creating a cryptographic representation of the content. This hash is then utilized in the invocation of the smart contract function, ‘CreateMessage’. The function call includes the Parent ID, which corresponds to the original request ID from Hospital (A)’s case, thereby linking the response to the initial request.
- **Finalizing the Blockchain Transaction**: The text of the response is digitally signed to ensure authenticity, integrity, and non-repudiation of the message. The signature process employs the ECDSA, a robust technique for creating verifiable digital signatures.
- **Adding to the Blockchain and Broadcasting**: The signed response is added to a new block within the blockchain. This block is timestamped and appended to the existing chain, maintaining the continuity and immutability of the data. Once this block is created, it is broadcast to all peers within the blockchain network, thus updating all participants about the ongoing consultation and the newly available information from Hospital (B).

This sequence of actions demonstrates the platform’s commitment to maintaining a secure and efficient communication channel for medical consultations. By utilizing blockchain technology, smart contracts, and decentralized storage solutions, the platform ensures that sensitive patient information is handled with the utmost care, allowing for transparent and secure professional collaboration.

Fig 10 illustrates the secure exchange of EHRs between hospitals within the blockchain-based medical consultancy platform. The figure depicts the sequence of steps taken by Hospital (A) to respond to a consultation offer from Hospital (B) and to securely transmit relevant patient EHRs. Thus, upon receiving a consultation offer, Hospital (A) accesses the Unified BC Portal for Medical Consultancy and retrieves the offer from the home page. Recognizing the intent to consult from Hospital (B), Hospital (A) proceeds to select the pertinent EHR associated with the case.

- **EHR Selection and Preparation**: Hospital (A) carefully selects the EHR pertinent to the case, ensuring that any personal patient information is anonymized to maintain privacy before uploading the document to the platform.
- **EHR Uploading and Hashing**: Hospital (A) then uploads the EHR to the IPFS, which returns a hash of the uploaded file. This hash serves as a unique identifier for the EHR.
- **Smart Contract Interaction**: Utilizing the smart contract function ‘CreateMessage’, Hospital (A) includes the hash of the EHR along with the Parent ID—the Consultancy Request and the public address for Hospital (B). This smart contract encapsulates the details necessary for the transaction and links it to the initial request for consultancy. To ensure secure access to the EHR, the hash or reference of the EHR is only shown to hospital (B),this is done through assigning the Public address of the hospital (B) to the encrypt variable.
- **Blockchain Storage and Sharing**: The transaction, containing the EHR’s hash, is stored on the blockchain. This action ensures the immutability and traceability of the EHR within the secure blockchain network.
- **Broadcasting to Network Peers**: Once the transaction is added to a new block and signed, it is broadcast to all peers within the Cancer Blockchain network. This process notifies all participants of the new data addition, though only Hospital (B) can access and read the EHR, ensuring confidentiality and secure access to patient information.

This process exemplifies the system’s capability to facilitate secure communication and data exchange between participating hospitals, leveraging blockchain technology to ensure the confidentiality and integrity of patient data while fostering collaborative medical consultation.

Fig 11 illustrates the workflow where Hospital (B), upon receiving the EHR from Hospital (A), commences the evaluation of the patient’s case. The steps are outlined as follows:

- **Case Review and Analysis**: Hospital (B) meticulously reviews the EHR provided by Hospital (A). This involves a detailed analysis of the case and the patient’s symptoms, leading to the development of a comprehensive medical report and plan.
- **Consultation Report Preparation**: After a thorough diagnosis and planning, Hospital (B) compiles the Consultation Report (CR), which includes their expert recommendations and proposed treatment plan for the patient’s case.
- **Secure Report sharing**: Prior to sharing, the CR is access restricted using the public key of Hospital (A), denoted as PU(A). This ensures that the report can only read by Hospital (A), maintaining patient confidentiality and secure communication.
- **Uploading and Hashing the Report**: The CR is uploaded to the IPFS, which generates a hash of the document, serving as a unique identifier for this specific communication.
- **Blockchain Transaction Creation**: Hospital (B) then invokes the smart contract function ‘CreateMessage’, incorporating the hash of the CR and specifying the Parent ID as the original Consultancy Request along with Hospital (A)’s public address in the encrypt variable. This creates a new transaction on the blockchain that securely records the exchange.
- **Storing Data on the Blockchain**: The transaction, now containing the encrypted CR, is added to a new block on the blockchain. This block is timestamped and securely linked to the existing chain.
- **Broadcasting the CR**: Finally, the new block with the CR is broadcast to all participants within the Cancer Blockchain network. Due to the access restriction, only Hospital (A) has the capability to access the content of the CR.

This process, as depicted in Fig 11, showcases the robust use of blockchain technology for secure and private communication between healthcare institutions. Smart contracts, and the IPFS for secure data management, the platform ensures that sensitive medical information is exchanged with integrity and confidentiality, facilitating a collaborative approach to patient care.

Fig 12 outlines the process for Hospital (C) to engage privately with Hospital (A) via the B-UMCS following the completion of a consultation that involved Hospital (B). So, Hospital (C) accesses the B-UMCS and navigates to the home page. Their objective review the CR shared on the case ‘1’, since it is only accessible to Hospital (A), Hospital (C) has to request it from the data owner, which is hospitak (A). This is done though the following:

- **Loading the Home page**: In the context of a recently completed consultation case, Hospital (C) expresses its intent to obtain detailed case information that was initially shared by Hospital (A) and subsequently consulted upon by Hospital (B)
- **Drafting a private request Messages**: To accomplish this, Hospital (C) composes a private message directed at Hospital (A), indicating the public key of Hospital (A) (PU(A)) as the message’s destination. This ensures that the message, upon being sent, remains confidential and accessible solely to Hospital (A).
- **Saving the private message on IPFS**: once The message is drafted, undergoes the standard procedure of uploading to IPFS, and hashing. The smart contract ‘CreateMessage’ function is called with the appropriate parameters, including the hash and the public address of Hospital (A) as the value assigned to the receiver attribute.
- **Storing the Data on Blockchain**: This message is then added to the blockchain as a new transaction, signed to maintain integrity and authenticity, and finally broadcast to the network. However, due to the rule (Receiver == PU(A)), only hospital (A) will be able to review the communication.

This process facilitates a secure and private channel for hospitals to request and share sensitive case details, further enhancing collaborative efforts in patient care within the network.

The process by which Hospital (A) receives and responds to private messages from Hospital (C) is delineated in Figs 13 and 14. It is demonstrated that Hospital (A), upon receipt of a communication from Hospital (C), initiates the retrieval of private messages through the execution of the smart contract function ‘FetchReceivedMessages’. Through this method, Hospital (A) identifies the specific message dispatched from Hospital (C). In response, Hospital (A) proceeds to restrict the access the CR using Hospital (C)’s public key, denoted as PU(C), ensuring the message’s confidentiality. Subsequently, the CR is uploaded to the blockchain, where it becomes a part of the immutable ledger, accessible only by Hospital (C). Thus, Figs 13 and 14 illustrate the steps for receiving and sending messages within the blockchain infrastructure. Furthermore, Hospital (A) has the capability to audit all sent messages, both private and public, by invoking the ‘FetchSentMessages’ function. This function allows Hospital (A) to oversee their communication history, contributing to a transparent and secure exchange of information within the medical consultancy blockchain network. This robust mechanism ensures that all exchanges between hospitals A and C remain secure, private, and verifiable at any given time (See Fig 15).

In the concluding phase of the communication protocol within the blockchain-based medical consultancy system, Hospital (C) employs a procedure analogous to the one depicted in Fig 13 to retrieve their incoming messages. By invoking the ‘FetchReceivedMessages’ function of the smart contract, Hospital (C) is able to access the list of messages directed to it. This function provides Hospital (C) with the ability to identify and securely download the CR that Hospital (A) has transmitted.

The security protocols established within the system ensure that when Hospital (C) initiates the download of the CR, it does so through a secure channel, maintaining the confidentiality and integrity of the patient’s data. The CR, encrypted and stored on the blockchain, can only be read by Hospital (C), as it is the intended recipient with the corresponding private key.

This final step exemplifies the secure and efficient exchange of medical consultation data facilitated by the system, demonstrating the practical application of blockchain technology in enhancing communication and collaboration among healthcare institutions.

## B-UMCS implementation

**Fig 16:**
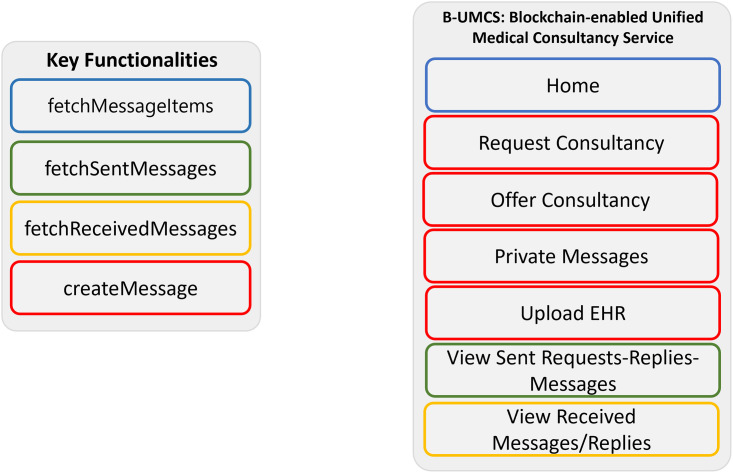
Smart contract and platform functions mapping.

This section delineates the comprehensive implementation details of the proposed B-UMCS. It explain the technical base and the methodological approach adopted for the system’s development. Furthermore, a thorough examination of the tools utilized in the realization of the blockchain infrastructure is presented. This encompasses a discussion on the deployment of Node.js as the runtime environment, Solidity for smart contract development, React for building user interfaces, and MetaMask for Ethereum blockchain interaction. The ensuing discourse gives a detailed look at the system’s technical setup and how its components work together. Additional elaboration on these technological tools is provided below:

- **Node.js**: Node.js serves as the cornerstone for server-side operations within our DApp ecosystem. As an open-source, cross-platform JavaScript runtime environment, Node.js simplifies asynchronous event-driven programming, making it an indispensable tool for the development of robust DApps. Its utility in this context extends to the seamless integration of Blockchain APIs and the facilitation of user interface interactions [53].
- **React**: The React library, known for its agility in building dynamic user interfaces, is instrumental in the system’s front-end development. React’s component-based architecture has shifted traditional web development paradigms by amalgamating HTML and CSS within JavaScript, a move that resonates with modern development workflows that prioritize modular, reusable code [54].
- **Solidity**: Tailored for the creation of smart contracts on blockchain platforms, Solidity is the language of choice for establishing the contract-centric backbone of our system. The implementation utilizes Solidity’s compatibility with both private and public blockchain networks, optimizing the deployment of smart contracts that encapsulate key operational data such as addresses and transaction histories [55].
- **MetaMask**: MetaMask, functioning as an Ethereum wallet, acts as the interface between the user’s browser and the Ethereum blockchain. It facilitates the execution of DApp transactions within the browser environment, providing a user-friendly platform for identity management and secure transaction verification [56, 57].
- **Unified Medical Consultancy Chain**: The platform is designed as a digital confluence point for medical specialists from diverse locations. It features a streamlined user interface with four primary tabs—Home, Request Consultancy, Sent Communications, and Received Messages—each corresponding to specific functions within the smart contract. This mapping is critical for achieving a cohesive and intuitive user experience, as visualized in Fig 16.

These technological components are carefully integrated into the proposed system to create a secure, efficient, and user-centric platform for medical consultancy services. The selection and application of each technology have been informed by its capacity to contribute to the overarching goal of enhancing the healthcare delivery process through advanced blockchain solutions.

### System functionalities

The operational flow of the B-UMCS is comprehensively depicted through the sequences in Figs 3–5. Following the registration process, Hospitals A, B, and C have successfully become integral members of the B-UMCS. These entities are actively engaged in the system, poised to seek and provide support, and committed to the collaborative sharing of knowledge and expertise.

Thus, to enable the interactive features of the B-UMCS, the system necessitates each participating hospital to register a distinct account within MetaMask. This integration is pivotal for linking the platform with the Ethereum blockchain. MetaMask, a prominent Ethereum wallet, operates within the browser as an extension, thereby providing a seamless interface for managing blockchain transactions and accounts.

**Fig 17:**
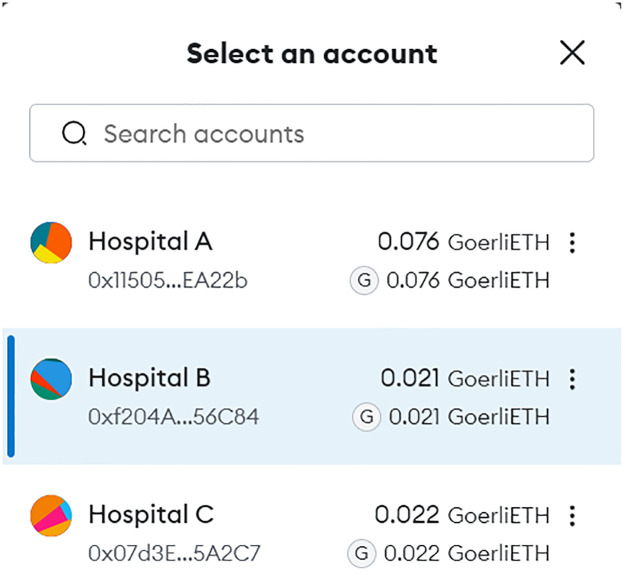
MetaMask account setup displaying testnet balances for Hospitals A, B, and C.

Fig 17 showcases a typical MetaMask account selection interface, presenting a list of hospital accounts that have been set up on the Ethereum test network, GoerliETH. For instance, Hospital (A) is represented with the account identifier 0x11505…EA22b’, reflecting a balance of 0.076 GoerliETH, indicative of the Ethereum available for test transactions. Similarly, Hospitals B and C are denoted with their respective unique account identifiers 0xf20A4…56C84’ and ‘0x07d3E…5A2C7’, each holding balances of 0.021 GoerliETH and 0.022 GoerliETH, respectively.

These account balances represent the ‘ether’ available to each hospital, facilitating the execution of smart contract functions such as posting consultation requests, sending and receiving messages, and sharing EHRs within the test environment. By establishing these accounts, hospitals ensure their readiness to engage with the blockchain-based service platform, effectively managing transactions related to consultancy services within the secure ecosystem of the Ethereum blockchain.

The utilization of the Goerli test network allows for a controlled and safe environment where hospitals can simulate the transactional functionalities of the system before live deployment, ensuring a thorough understanding of the processes and interaction dynamics without expending real currency. This setup is essential for verifying the system’s operations and ensuring a flawless user experience upon the transition to the main Ethereum network for actual service implementation.

Consequently, by leveraging the Ethereum blockchain through Metamask, the platform guarantees the immutability and traceability of transactions, which are essential for maintaining the integrity and trustworthiness of the system’s operations. This synergy between the platform’s architecture and the blockchain infrastructure underpins the robust functionalities of the B-UMCS, enabling a decentralized yet coordinated approach to medical consultancy services.

In the seamless continuum of the B-UMCS’s development, the creation of MetaMask accounts for each participating hospitalRepresents an important turning point, Setting the stage for features that are crucial to the platform’s function.. The subsequent subsections will expound upon these functionalities in detail: Accessing the Home Page, Requesting consultancy, Offering Consultancy, Sharing EHRs, Distributing Medical CRs, as well as the details of sending and receiving private messages, and reviewing Sent and Received Messages. Each function is meticulously architected to leverage the decentralized capabilities of blockchain technology, ensuring secure, transparent, and efficient interactions among healthcare providers. This cogent array of features encapsulates the essence of the system’s design—intuitive user interfaces that facilitate not only the navigation and management of medical data but also underscore the collaborative ethos at the heart of the platform. Through this integration, the platform stands as a testament to the convergence of technological innovation and medical collaboration, fostering a robust environment for medical consultancy and patient-centric care.

#### Accessing the home page

**Fig 18:**
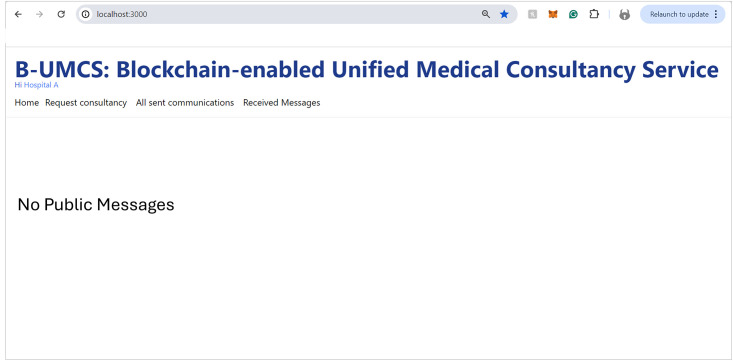
Hospital (A) accesses the B-UMCS.

Upon successful authorization, Hospital (A) gains entry to the B-UMCS. The Home page, which is the default landing interface upon login, presents a comprehensive view of all active and historical consultation requests. This feature is triggers the Algorithm 1, which delineates the smart contract function responsible for aggregating all consultations and publicly accessible responses (See Fig 18). The figure illustrates the user interface, clearly identifying the active user in the top-left corner; in this instance, it denotes Hospital (A) as the currently active user, thus personalizing the user experience and ensuring that the relevant user’s activities and messages are conveniently accessible.

**Algorithm 1** FetchMessageItems

**Output**: Returns an array of public consultations and replies that are visible to the querying hospital.

1: **procedure** FetchMessageItems

2:  *itemCount* ← Current(_*messageIds*)

3:  *publicItemCount* ← 0

4:  *currentIndex* ← 0

5:  **for** *i* = 0 **to** *itemCount* − 1 **do**

6:   **if** IsPublic(*idToMessageItem*[*i* + 1] **then**

7:    *publicItemCount* ← *publicItemCount* + 1

8:   **end if**

9:  **end for**

10:  **DECLARE** items **as** array **of** MessageItem **with size** publicItemCount

11:  **for** *i* ← 0 **to** itemCount—1 **do**

12:   **if** idToMsgItem[*i* + 1].isPublic = true **then**

13:    *currentId* ← *i* + 1

14:    *currentItem* ← idToMsgItem[*currentId*]

15:    *items*[currentIndex] ← *currentItem*

16:    **if** *items*[currentIndex].attachementURI ≠ “” **AND NOT**(msg.sender = idToMsgItem[*i* + 1].encrypt **OR** idToMsgItem[*i* + 1].encrypt = address(0)) **then**

17:     *items*[currentIndex].attachementURI ← “Restricted”

18:    **end if**

19:    currentIndex ← currentIndex + 1

20:   **end if**

21:  **end for**

22:  **return** items

23: **end procedure**

The **FetchMessageItems** 1 Algorithm is responsible for retrieving an array of all the public messages. First it initiates a variable to count the Number of Public Message items. The function creates a loop function and retrieve message items, the items either can be the consultation request or their replies from the blockchain. Then, it checks the (isPublic)variable included within the data stored on the blockchain **if (isPublic == True)**, then this message item will be included within an array of public messages that is later returned as an output to be displayed on the home page. Then, the function also checks the **“AttachementsURI”** of each message item if it is not null And The sender’s public address is the same public Address in the encryption variable, or the public address of the encryption variable is not “0”, then Attachment IPFS hash will be restricted.

#### Requesting consultancy

Faced with a shortage of oncology specialists and an increasing number of cases, Hospital (A) turns to the B-UMCS to solicit external expertise. To initiate a request for assistance, the hospital navigates to the “*Request Consultancy*” tab, employing the procedures outlined in Algorithms 2 and 3.

The **CreateMessge** 2 Algorithm creates a new message and associate it with a unique ID **NewMsgID** and sets its metadata which is the IPFS hash returned from storing the message request on the IPFS. The Function calls the function **mint** which is used to create a new request message (Digital Asset) and assign it to a given public address, which in this case is the **Sender** variable. The **SetTokenURI** is used to set a new URI or reference to the new request message, to allow retrieving the request message when needed. Then, a **sendMessageItem** private function is called, including the updated variables as an input to the functions (the Message unique ID, the receiver Address, the Parent ID of the message, the address used to encrypt the CR’s referense, and the Attachment IPFS hash). Finally, the Message ID is returned.

The Algorithm **SendMessage** 3 is used to Handles the creation and storage of message items, the Functions take a variable as an input, The Message ID, The Public Address of the receiver, The Case ID (ParrentId), The Public Address needed for an referense encryption, The IPFS hash of Attachment (If there is an attachment). The Function checks the Receiver variables, if it’s “0” meaning it is not sent to a particular Public Address. It will be flagged as a public Message (isPublic = True), if the Variable hold a specific Address, it is then flagged as a private Message (isPublic = False). Finally, the “MessageItemSent” Event is emitted including the 7 variables to create the Message transaction, Events are components of contracts Which When triggered, they record their arguments in the transaction log, a specific data structure on the blockchain.

**Algorithm 2** Create Message Function

**Input**: messageURI, address of the receiver, parentId

**Output**: providing the message IPFS Hash, receiver’s address, parent ID (if any), and encryption address (if applicable). The function returns the new message ID.

 1: **procedure** CreateMessage(*messageURI*, *receiver*, *parentId*, *encrypt*, *attachmentURI*)

 2:  MsgID ← MsgID + 1

 3:  newMsgID ← MsgID

 4:  _mint(msg.sender, newMsgID)

 5:  _setTokenURI(newMsgID, messageURI)

 6:  sendMessageItem(newMsgID, receiver, parentId, encrypt, attachmentURI)

 7:  **return** *newMessageId*

 8: **end procedure**

**Algorithm 3** Send Message Function

**Input**: MsgID, receiver, parrentId, encrypt, attachementURI.

**Output**: None.

 1: **procedure** SendMessageItem(*MsgID*, *receiver*, *parentId*, *encrypt*, *attachmentURI*)

 2:  **if** receiver = address(0) **then**

 3:   publicItems ← publicItems + 1

 4:   idToMsgItem[msgID] ← MsgItem(MsgID, parentId, msg.sender,

 5:   receiver, true, encrypt, attachmentURI)

 6:  **else**

 7:   idToMsgItem[MsgID] ← MsgItem(MsgID, parentId, msg.sender, receiver, false, encrypt, attachmentURI)

 8:  **end if**

 9:  emit MessageItemSent(MsgID, parentId, msg.sender, receiver, receiver = address(0), encrypt, attachmentURI)

 10: **end procedure**

This dual-algorithm process facilitates both broad and targeted communications, underpinning the platform’s capacity to adapt to the diverse needs of its users. The system’s architectural design, coupled with the blockchain’s immutable ledger, ensures that every consultancy request is traceable, secure, and handled with the utmost efficacy.

**Fig 19:**
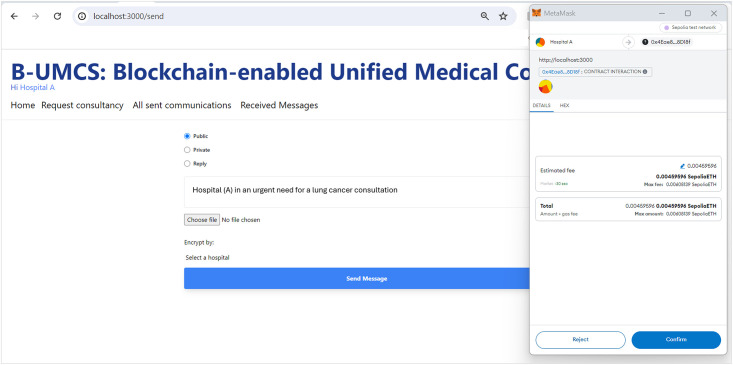
Hospital (A) requests a medical consultation.

Fig 19 shows the user interface for consultancy requests, enabling the hospital to determine the audience of the message. The system offers the option to disseminate the message either publicly or privately, or as a reply to an ongoing consultation thread. In the presented scenario, Hospital (A) opts for the “Public” setting, seeking wide engagement for their request.

**Fig 20:**
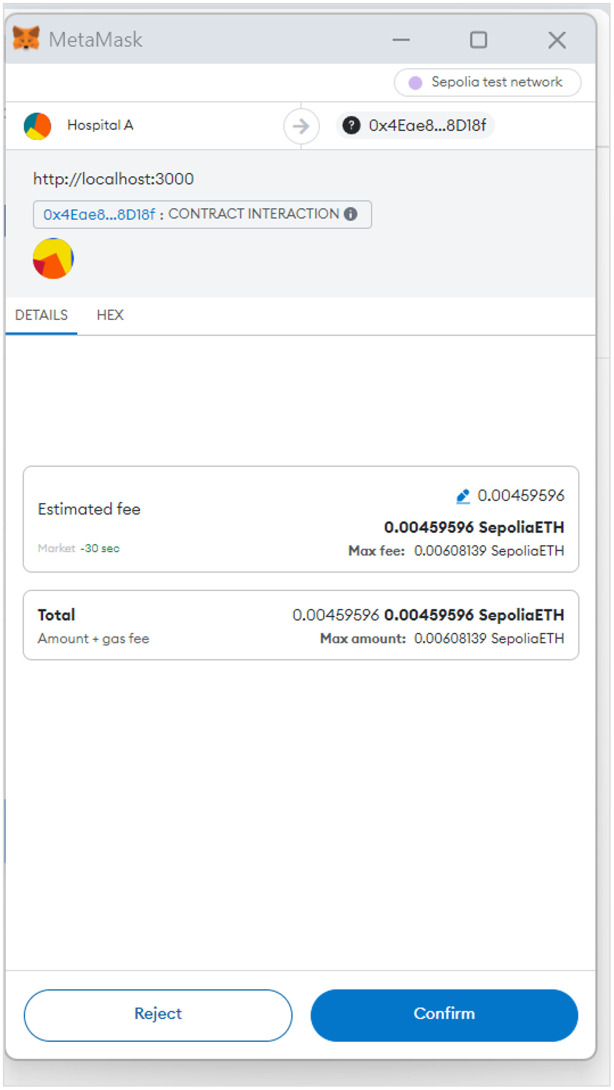
Contract transaction details.

Upon selecting the message content for broadcast, as depicted in Fig 19, Hospital (A) engages the “Send Message” feature. This action triggers the MetaMask wallet interface, which serves as the gateway to the Ethereum blockchain’s backend processes. The MetaMask prompt delineates the transaction’s particulars, including the Ethereum account address, the stipulated gas limit which is the fee required for successfully complete a transaction, the actual gas consumption, which is the fee, and the corresponding cost associated with this specific transaction. It then presents the user with the option to either authenticate or negate the contract interaction, ensuring that the user retains control over the transaction confirmation process. Fig 20 provides an insightful view into the MetaMask transactional interface, illustrating the critical steps of user verification required for successful transaction execution on the blockchain.

This step is vital in preserving the security and integrity of the system, as it requires explicit user consent for any blockchain transaction, thereby reinforcing the trustworthiness of the B-UMCS. It exemplifies the seamless integration of user interface actions with the underlying blockchain operations, enhancing the platform’s user experience while upholding the stringent security measures inherent to blockchain technology.

**Fig 21:**
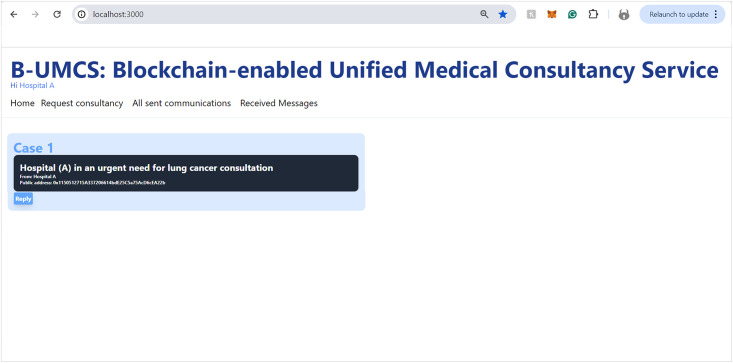
Display format for consultation requests and replies on the proposed platform.

In the B-UMCS platform, each consultation request or reply is systematized into a cohesive format, comprising five essential components for straightforward navigation and comprehension. Fig 21 exemplifies this format:

- **Case Number**: Prominently displayed at the commencement of the entry, this numerical identifier correlates to each new public consultation. It is designed to facilitate easy tracking and referencing within the platform’s case management system.
- **Message**: Positioned as the foremost element within the entry, the message succinctly encapsulates the consultation’s core appeal or response, intended for dissemination to peers on the blockchain.
- **Sender Name**: This element serves to attribute the communication to its originator, displaying the name of the hospital responsible for the consultation request or reply.
- **Public Address**: It reveals the blockchain public address of the sender, a unique digital identifier within the blockchain network, ensuring transparency and traceability. The public address is a shortened and hashed form of the public key.
- **Attachment**: This component becomes visible only when there is an inclusion of additional documentation, such as EHR or Medical CR, tied to the message.

These components are intricately woven into the platform’s interface to ensure a high degree of clarity and accessibility for all participants, thereby enhancing the overall efficacy of the consultation process. Therefore, this standardized representation of cases not only streamlines the consultative exchange but also fortifies the process with a level of detail necessary for comprehensive peer review and collaborative decision-making.

Fig 23 illustrates the dialog where Hospital (B) is formulating a response to the initial request, clearly indicating their reply pertains to case number 1. This sequence is underpinned by the smart contract functions enumerated in Algorithms 2 and 3, which operationalize the communication. The parameters for the recipient’s address and the parent message identification are established as follows, with **ParentID** equating to the original case number, thus interlinking the subsequent communication with the initial request:

ParentID=1

#### Offering consultancy

**Fig 22:**
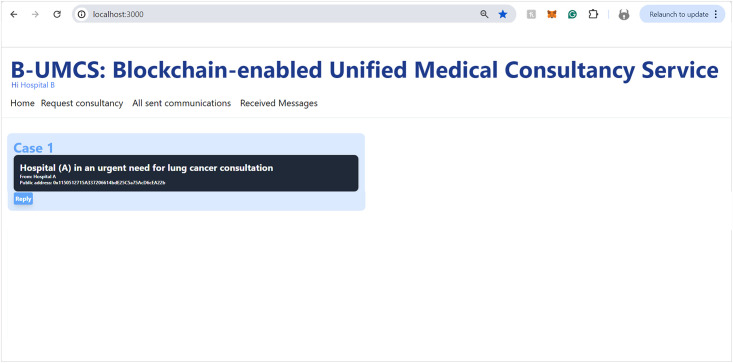
Visibility of consultation request to Hospital (B) within the network.

**Fig 23:**
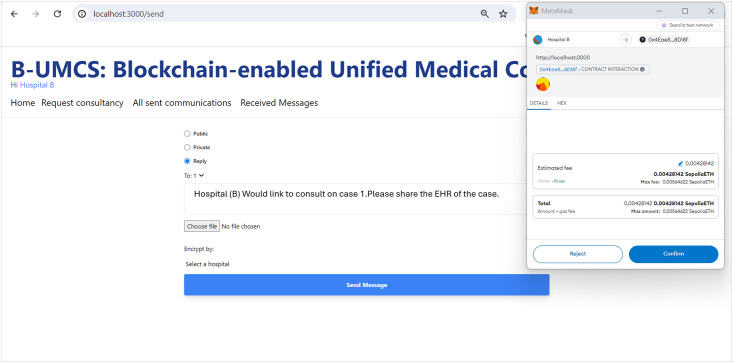
Hospital (B) formulates a Reply to the consultation request.

Upon the dissemination of a consultation request by Hospital (A), Hospital (B) recognizes the request and decides to offer their expertise to the case. To initiate this process, Hospital (B) engages with the consultation announcement as exhibited in Fig 22, by selecting the “Reply” option provided therein. This action transports Hospital (B) to the dedicated reply interface, which is depicted in Fig 23. Here, Hospital (B) articulates their willingness to consult on the case by requesting additional specifics concerning the patient’s condition and associated EHR.

#### Sharing the EHR

**Fig 24:**
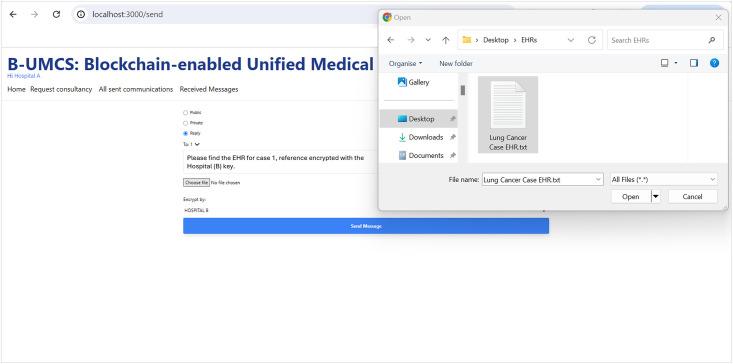
Hospital (A) uploads the EHR to the platform to be then saved on IPFS.

Following the reception of Hospital (B)’s solicitation for additional details, Hospital (A) responds by securely transmitting the EHR through the platform. This is achieved by limiting the access the EHR by only restricting the access of the IPFS hash to only Hospital (B) by using it’s public key (PU(B)). Fig 24 depicts Hospital (A) in the process of uploading the EHR. This step involves selecting the pertinent file from their local device and restricting the access, to ensure for confidentiality and integrity of the patient’s file. Hospital (A) proceeds to send the message, confident in the secure delivery of the sensitive health record exclusively to Hospital (B).

**Fig 25:**
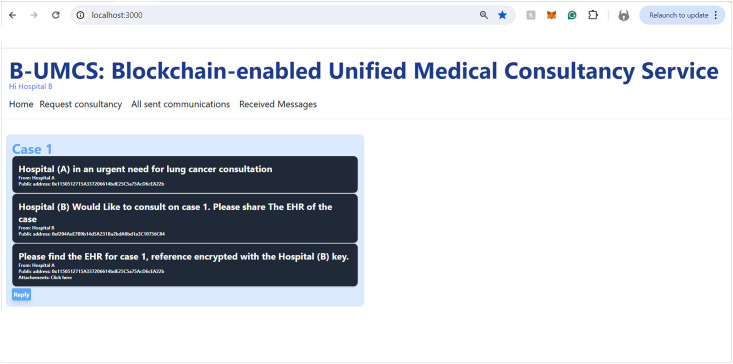
Exclusive access granted to Hospital (B) for reviewing the EHR, demonstrating the efficacy of the restriction protocol.

**Fig 26:**
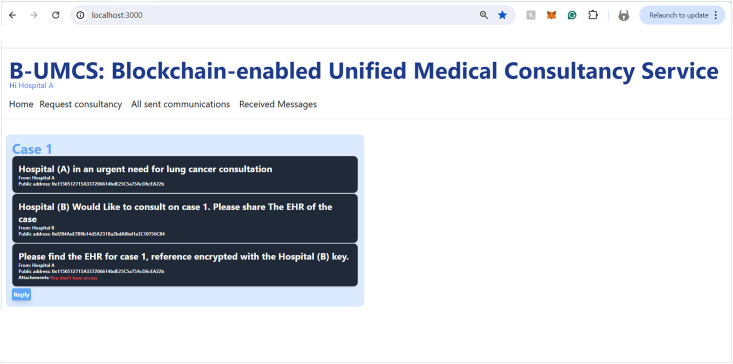
Restricted access notification for Hospital (A), highlighting the platform role in preserving data privacy.

**Fig 27:**
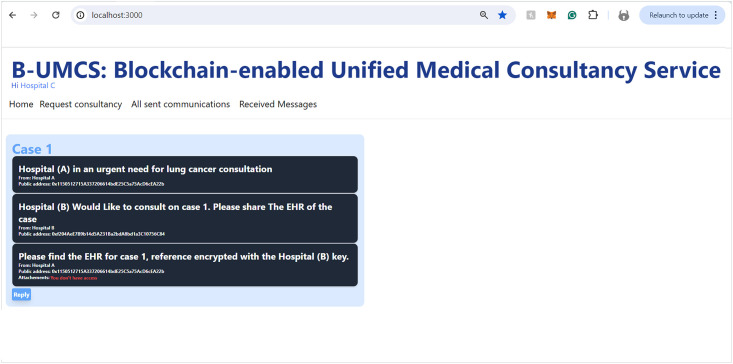
Hospital (C) encounters access restrictions, reinforcing the secure and targeted dissemination of medical data.

Upon Hospital (A) initiating the transfer of the EHR, the secure exchange between the hospitals is displayed across the B-UMCS interface. Hospital (B), possessing the private key, is thereby granted exclusive access to review the pertinent EHR. Figs 25–27 collectively narrate the interface’s updates, presenting a coherent sequence of interactions between Hospitals A and B. Particularly, Fig 25 validates that Hospital (B), upon interaction with the encrypted EHR referense, is provided the option to review and retrieve the document. In stark contrast, Hospitals A and C are presented with a clear notification stating “You don’t have access,” underscoring the rigorous enforcement of data confidentiality facilitated by the platform’s cryptographic protocols.

Algorithm 4 elucidates the mechanism underlying the insurance of authorization to provide the IPFS hash of EHRs within the smart contract’s framework. The algorithm is methodically designed to authenticate the presence of an attachment within the current message. Should an attachment be confirmed, and provided the encrypting public address diverges from the sender’s or remains unspecified, the attachment URI is accordingly adjusted to a “Restricted” status. This safeguarding measure ensures that only the intended recipient, armed with the appropriate public key, is authorized to access the sensitive EHR information, thereby preserving patient confidentiality and maintaining the integrity of the medical data exchanged across the blockchain network.

**Algorithm 4** Checking the Authorization of the attachment’s IPFS hash

1: Assign *currentItem* to *items*[*currentIndex*]

2: **if** the hash of *items*[*currentIndex*].*attachmentURI* is not empty **and**

3:  (the sender is not equal to *idToMessageItem*[*i* + 1].*encrypt* **or**

4:  *idToMessageItem*[*i* + 1].*encrypt* is the null address) **then**

5:  Set *items*[*currentIndex*].*attachmentURI* to “Restricted”

6: **end if**

**Fig 28:**
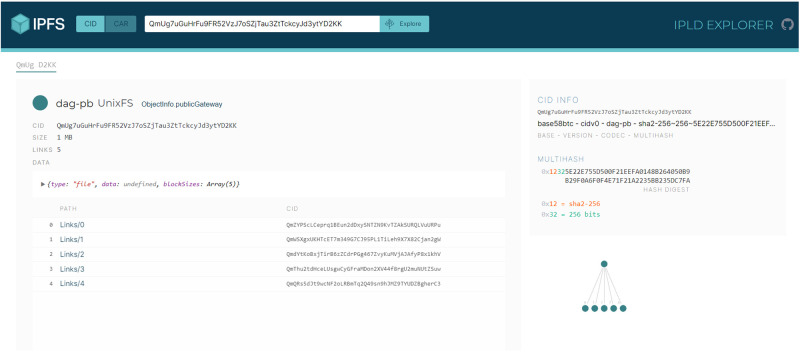
Illustration of an EHR’s secure storage and reference within the IPFS, highlighting the data’s decentralization and immutability.

Fig 28 presents a visual representation of the IPFS storage mechanism for a medical record. The structure illustrated denotes the distributed nature of IPFS, a content-addressable, peer-to-peer hypermedia distribution protocol. In this depiction, we observe a file characterized by a unique Content Identifier (CID), which encapsulates the file’s hash, providing an immutable reference within the IPFS network. The multihash details reveal the security and integrity of the file storage, with the SHA-256 cryptographic hash function serving as a cornerstone of this robust system. IPFS saves files on various nodes throughout its network. It utilizes a data structure known as a DAG to arrange and handle connections to nodes of data, where each node is a segment of a document, and each node contains (a hash of the node itself and links to other nodes), ensuring that the document remains intact and verifiable across the decentralized network. Such a system guarantees that the document’s authenticity and integrity are maintained without reliance on a single point of failure, embodying the principles of redundancy and resilience inherent to blockchain technologies.

The architecture of the IPFS presents a multi-tiered linkage system where each node represents a block of data. Within this framework, the data is not stored in a singular location but rather distributed across a network, enhancing resilience and preventing single points of failure. The versatility of this system allows healthcare providers to interact with a robust data exchange platform, where the security of sensitive information is not a matter of concern but a given assurance. So, in the broader context of blockchain technology and its applications in healthcare, the utilization of IPFS represents a paradigm shift towards a more open, interoperable, and secure framework for health information exchange. The implications for patient care, research, and medical collaboration are substantial, as such systems enable the seamless, secure, and swift sharing of critical health data across institutional and geographic boundaries.

#### Sharing the medical CR

**Fig 29:**
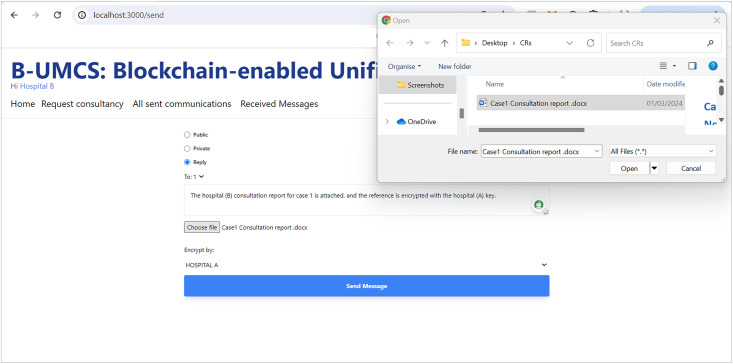
Hospital (B) engaging in the upload the CR to the platform to be then saved on IPFS.

**Fig 30:**
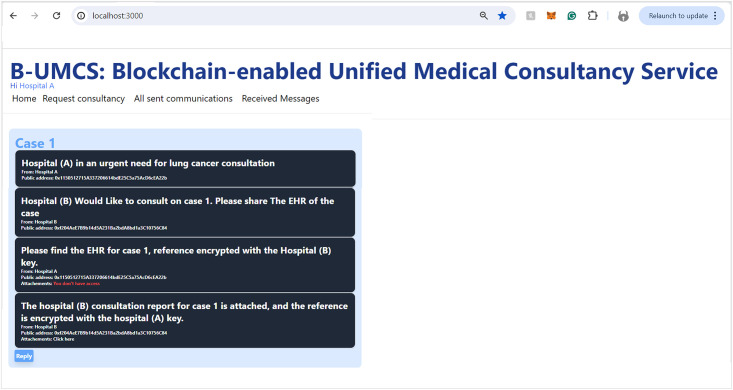
Hospital (A) authorized to access the CR.

Subsequent to the reception of the EHR by Hospital (B), a diagnostic process is initiated, culminating in the creation of a medical CR. Post-diagnosis, Hospital (B) is tasked with disseminating the CR via the platform. In alignment with preserving consultation secrecy and ensuring the confidentiality of the report, it is safeguarded through restricting the access using the public address of Hospital (A). Depicted in Fig 29 is the procedure of Hospital (B) uploading the CR and using the PU(A) to restrict the acquiring of the IPFS Hash only to hospital (A) Address. Conversely, Fig 30 delineates the exclusive accessibility of Hospital (A) to the CR bequeathed by Hospital (B), precluding any potential access by tertiary hospitals. This exclusive exchange is operationalized through the invocation of Algorithm 4, as delineated in the smart contract, which stipulates the mechanisms for both uploading and restricting the access to the the CR’s IPFS hash on the designated platform. Upon receipt of the CR, Hospital (A), through the automated recognition of PU(A), is facilitated to retrieve the document.

#### Sending of private messages

**Fig 31:**
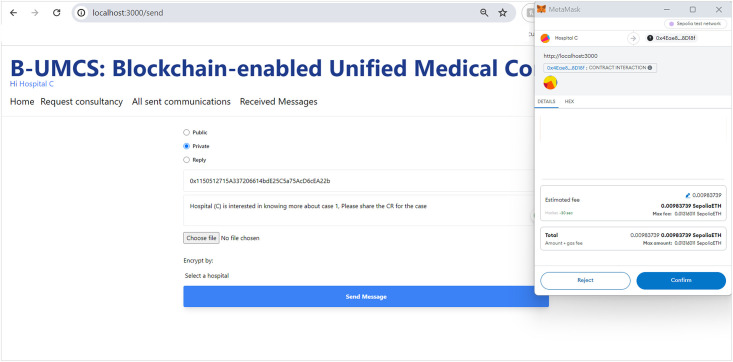
The process of Hospital (C) sending a private message to Hospital (A) for the CR.

Hospital (C) expresses a particular interest in Case 1 and desires to examine the CR pertinent to the case. In pursuit of this information, Hospital (C) commences a private communication channel with Hospital (A) to request the CR. Fig 31 depicts the user interface through which Hospital (C) initiates the private message to Hospital (A). In the interface, the communication type “Private” is explicitly selected, as indicated by the activated radio button. The interface necessitates the entry of the recipient’s Public (PU) address; in this instance, the Public address of Hospital (A). The interaction triggers the execution of the smart contract functions: “***CreateMEssgae()***” as referenced in Algorithm 2, and “***SendMessgae***” as indicated in Algorithm 3. The Address Receiver parameter is consequently set to the Public address of Hospital (A) (PU(A)) to facilitate the secure and private transmission of the message.

#### View sent messages

**Fig 32:**
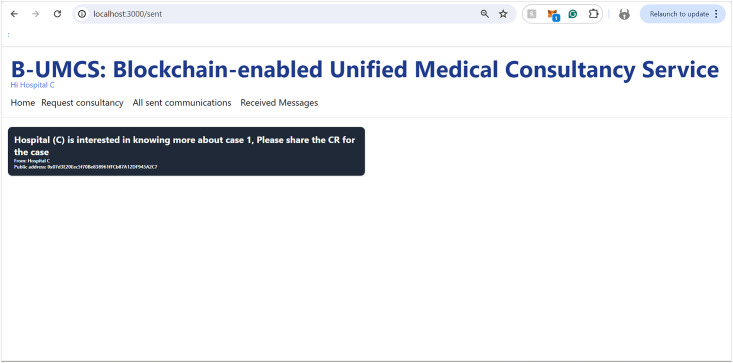
Interface for Hospital (C) to view all communications dispatched from their end.

Hospital (C) possesses the capability to review messages previously transmitted by navigating to the “All Sent Communications” tab. This specific interface element consolidates and displays all forms of communications—both private and public—originating from the user. Fig 32 provides a visual representation of the user interface displaying Hospital (C)’s sent communications. The functionality underpinning this feature is encapsulated in Algorithm 5, which delineates the procedure “***FetchSentMessages()***” for retrieving the sent messages.

The **FetchSentMessages** Retrieves an array of all messages sent by the current hospital. The function checks all message items within the blockchain. First a counter is incremented to count how many message items have the same public address in the *“Sender”* variable that is equal to the current hospital. Then a new temp array is created named *“items”*, then a new loop with the length of the counter is set to go through the blockchain data. Retrieve each message item’s data, checks the sender variable if it is equal to the current hospital, then the message item is then appended to the new array *“items”*. Finally it also checks the restriction over the IPFS hash, if it not empty and the current hospital is not the user, or the encryption variable holds the address zero, then it is restricted.

**Algorithm 5** Fetch Sent Messages

**Input**: None.

**Output**: Array Of all sent messages.

 1: **function** FetchSentMessages

 2:  *totalItemCount* ← CurrentMessageCount

 3:  *itemCount* ← 0

 4:  *currentIndex* ← 0

 5:  **for** *i* ← 0 **to** *totalItemCount* − 1 **do**

 6:   **if** Sender(*idToMsgItem*[*i* + 1] == CurrentSender **then**

 7:    *itemCount* ← *itemCount* + 1

 8:   **end if**

 9:  **end for**

 10:  *items* ← NewMessageItemArray(*itemCount*)

 11:  **for** *i* ← 0 **to** *totalItemCount* − 1 **do**

 12:   **if** Sender(*idToMsgItem*[*i* + 1] == CurrentSender **then**

 13:    *currentId* ← *i* + 1

 14:    *currentItem* ← GetMessageItem(*currentId*)

 15:    *items*[*currentIndex*] ← *currentItem*

 16:    **if** NotEmpty(*items*[*currentIndex*].*attachmentURI*) **and**

 17:  **not** (CurrentSender == Encryptor(*idToMsgItem*[*i* + 1] **or**

 18:  IsEncryptorNull(*idToMsgItem*[*i* + 1]) **then**

 19:     *items*[*currentIndex*].*attachmentURI* ← “Restricted”

 20:    **end if**

 21:    *currentIndex* ← *currentIndex* + 1

 22:   **end if**

 23:  **end for**

 24:  **return** *items*

 25: **end function**

#### View received messages

**Fig 33:**
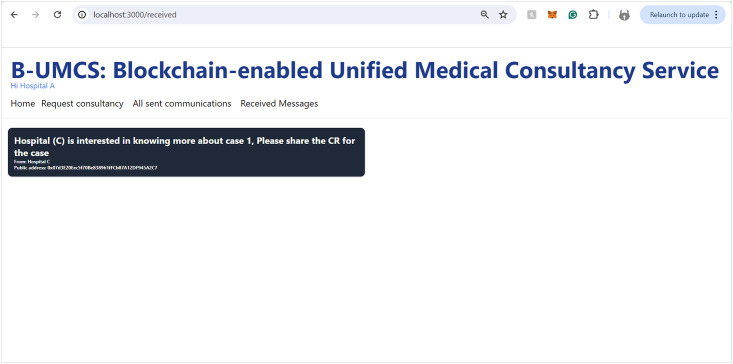
Hospital (A) views the received messages.

**Fig 34:**
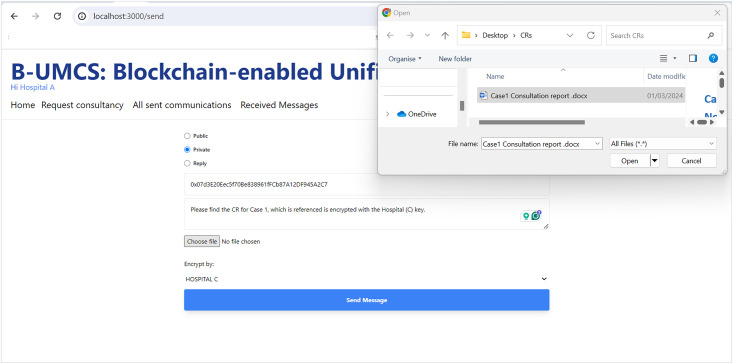
Hospital (A) sends the CR to Hospital (B) privately.

Upon sending a private message to Hospital (A), Hospital (C) awaits a response. Hospital (A), in due course, accesses the “Received Messages” tab within the platform to review any direct communications. Fig 33 displays the interface where only Hospital (A) can view messages intended for them, ensuring privacy. Subsequent to viewing the private message from Hospital (C), Hospital (A) is to reply with the CR, encrypted using Hospital (C)’s public key (PU(C)), and transmitted privately to the specified address (see Fig 34). This sequence of secure message exchange is underpinned by Algorithm 6, as outlined in the smart contract, which delineates the process of retrieving private messages.

**Algorithm 6** Fetch Received Messages

**Output**: Array of messages that the hospital has received privately.

 1: **function** FetchReceivedMessages

 2:  *totalItemCount* ← Current(_*messageIds*)

 3:  *itemCount* ← 0

 4:  *currentIndex* ← 0

     ▹ Count received messages

 5:  **for** *i* = 0 **to** *totalItemCount* − 1 **do**

 6:   **if** *idToMsgItem*[*i* + 1].*receiver* == msg.sender **then**

 7:    *itemCount* ← *itemCount* + 1

 8:   **end if**

 9:  **end for**

 10:  *items* ← new MessageItem array of size *itemCount*

    ▹ Populate array with received messages

 11:  **for** *i* = 0 **to** *totalItemCount* − 1 **do**

 12:   **if** *idToMsgItem*[*i* + 1].*receiver* == msg.sender **then**

 13:    *currentId* ← *i* + 1

 14:    *currentItem* ← *idToMessageItem*[*currentId*]

 15:    *items*[*currentIndex*] ← *currentItem*

    ▹ Restrict attachment URI under certain conditions

 16:     **if** hash of *items*[*currentIndex*].*attachmentURI* ≠ empty **and**

   (msg.sender ≠ *idToMessageItem*[*currentId*].*encrypt* **and**

   *idToMessageItem*[*currentId*].*encrypt* ≠ null address) **then**

 17:     *items*[*currentIndex*].*attachmentURI* ← “Restricted”

 18:    **end if**

 19:    *currentIndex* ← *currentIndex* + 1

 20:   **end if**

 21:  **end for**

 22:  **return** *items*

 23: **end function**

The **FetchReceivedMessages** function works similar to the fetch Sent Messages, retrieves an array including all received messages to the current hospital, it first go through all message items within the blockchain, and checks if the *receiver* variable value is equal to the current hospital’s public address, if yes, it includes it to a newly declared array called *Items* that is then retrieved. It also checks the attachment restrictions if the IPFS hash is not null and the current sender’s PU is not equal to one of the messages encrypt variable’s value. Or if the encrypt variable’s value is equal to zero, then the attachment IPFS hash is then restricted.

## Discussions, in-depth analysis, and real-world examples

**Fig 35:**
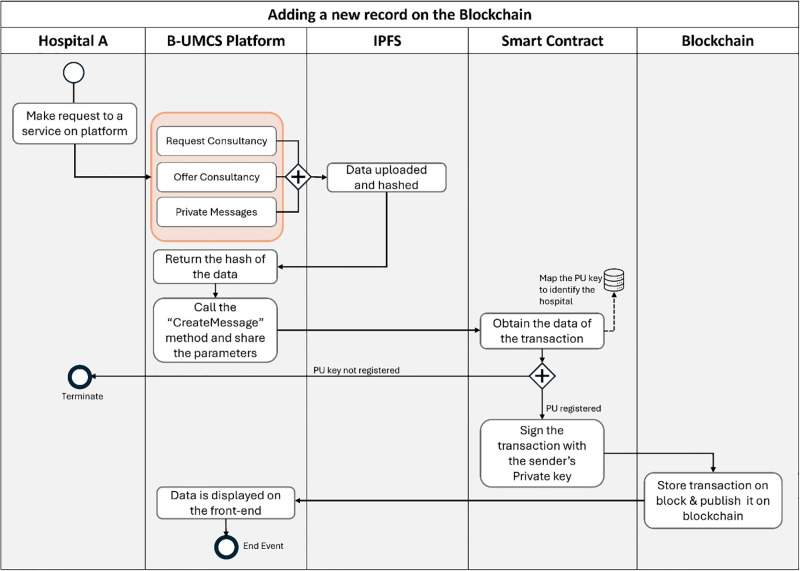
Process model illustrating the data flow for adding records on blockchain.

**Fig 36:**
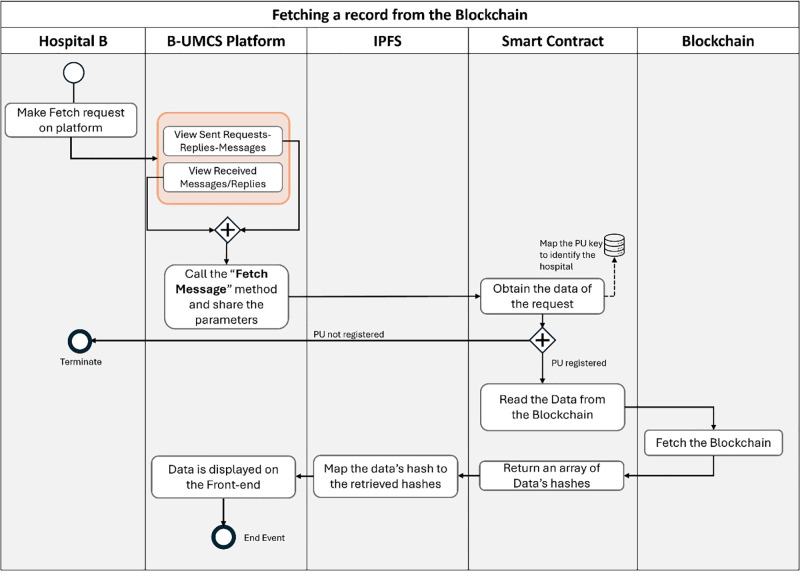
Process model illustrating the data flow for fetching data on the blockchain.

The B-UMCS is built on a robust blockchain infrastructure, leveraging Ethereum’s capabilities for secure and transparent transactions. Consequently, the B-UMCS architecture is designed and implemented to facilitate secure and efficient medical consultations among healthcare providers. The system’s core components include smart contracts, which automate processes and ensure data integrity, and the IPFS for decentralized data storage. Figs 35 and 36 elaborate more on the integration of blockchain by providing detailed process models of the data flow within the B-UMCS system. These models illustrate how records are added to the blockchain and how data is retrieved, ensuring secure and efficient transactions and consultations.

Fig 35 illustrates the data flow for making a transaction on the blockchain within the B-UMCS system. A process starts when a hospital triggers the B-UMCS system by either requesting a consultation (as detailed in Fig 6), offering a consultation (as shown in Fig 9), or sending private messages to another hospital (as represented comprehensively in Fig 12). In other words, the service request can change based on the parameter’s values. For example:

- If the (Receiver == null) && (ParentID == 0) && (IsPublic == true), then this means the service type is requesting a consultancy.
- If the (Receiver == Not null) && (ParentID == 1) && (IsPublic == true), then this leads to a service type of offering a consultation/reply, which targets a specific receiver.
- If the (Receiver == Not null) && (ParentID == 0) && (IsPublic == false), then this is considered a private messaging request service.

Upon selecting the request, the IPFS API is called to store the request and its parameter’s values (Sender, Receiver, ParentID, IsPublic,..etc). The IPFS subsequently stores and hashes the data, returning a hash of the stored data to the front-end to be used as a parameter while invoking the smart contract’s method (as detailed in Algorithms 2). The smart contract then ensures a secure transaction by conducting several authentication steps to verify the hospital’s identity. Finally, the transaction is signed with the hospital’s private key to ensure the authenticity and integrity of the data. Then, the data is added as a record in the block and dispatched on the blockchain for other hospitals within the system to review it. If EHRs are shared, they will be encrypted using the public key of the hospital that is interested in offering consultancy to ensure their confidentiality.

Fig 36 shows the reverse data flow in the B-UMCS system, which usually starts with the hospital fetching the received messages (as shown in detail in Fig 13) or fetching the sent requests that have been sent by the current hospital (as illustrated in Fig 15). These two service requests are similar in action but different in parameters. For example:

- If the requested fetch messages parameters are (Receiver == the current hospital’s public key) && (IsPublic == false) && (ParentID == 0), then this means the current hospital is trying to review the received message from other hospitals.
- If the requested fetch messages parameters are (Sender == current hospital’s public key) && the (receiver == Not null) && (IsPublic == false) && (ParentID == 0), then this indicates that the requested service is reviewing the sent messages by the current hospital.

After selecting the request, the system invokes the appropriate smart contract method for fetching the messages (as detailed in Algorithm 1). The smart contract ensures the security and authenticity of the current hospital’s identity, then it fetches all the blocks within the blockchain that match the appropriate parameters shared with the smart contract. An array of message hashes is retrieved from the blockchain and sent to the IPFS API to map the retrieved array of hashes with the current data hashes on IPFS. The data is then retrieved and displayed on the front-end of the B-UMCS.

In the following paragraphs, we provide detailed discussions, in-depth analyses, and examples of the key components of the B-UMCS. These examples assume several doctors from different hospitals are involved. Thus, in our examples, Dr. Smith is affiliated with Hospital A, Dr. Jones with Hospital B, Dr. Lee with Hospital C, and Dr. Patel with Hospital D. The examples illustrate the inter-hospital collaborations facilitated by the B-UMCS system, showcasing how doctors from different hospitals can securely share and consult on medical cases.

**Example (1)**: Dr. Smith from Hospital A needs a second opinion on a complex lung cancer case. Dr. Smith logs into the B-UMCS platform and submits a consultation request to get more insights from the experts in other hospitals registered in the B-UMCS. Both Dr. Jones from Hospital B and Dr. Patel from Hospital D received a notification of this consultation request as their hospitals have identified lungs cancer diseases as one of their key specialties. After checking the case, Dr. Jones decides to offer a consultation service. He requests more detailed medical reports, which would be sent encrypted only to him, to ensure the case’s privacy and information security.

**Example (2)**: Dr. Lee from Hospital C, a specialist in cardiology, logs into the B-UMCS platform and submits a consultation request through the user interface to a renowned cardiologist at Hospital D. The B-UMCS system allows Dr. Lee to send this direct message to Hospital D, with the option to make the consultation request visible to all or keep it private and accessible only to the concerned cardiologist at Hospital D.

**Example (3)**: The emergency unit at Hospital B has received a critically injured car accident victim. The X-ray images and test results reveal multiple severe injuries in various parts of the body, some of which fall outside Hospital B’s specialties. Consequently, Hospital B decides to utilize the B-UMCS system to send these anonymized images and results to other hospitals for specialized medical advice regarding the case.

The examples above are just a few instances highlighting the importance of the services the proposed B-UMCS system can offer to the medical sector. There are countless other scenarios where such a system would be invaluable. Below, we present detailed steps for one of the examples (Example 1) to illustrate the B-UMCS system’s process flow, highlighting the interaction between front-end and back-end components and utilizing blockchain and IPFS technologies.

**Step 1: Hospitals Registration** (Figs 3–5): This step describes the registration process for hospitals in the B-UMCS system, including submission of information, review by the MOH, and integration into the system.

1. **Registration by a Hospital** (Fig 3)
  - Hospitals A-D access the B-UMCS registration portal and provide necessary information such as hospital name, email address, and areas of medical specialty.
2. **Review by Ministry of Health (MOH)** (Fig 4)
  - The MOH reviews the application, evaluating the hospitals’ credentials and technological readiness.
3. **Approval and System Integration** (Fig 5)
  - Upon approval, the Hospitals A-D are integrated into the B-UMCS system, enabling access to consultation services.

**Step 2: Consultation Request Initiation** (Figs 6 and 19–21): This step details how a healthcare provider initiates a consultation request through the B-UMCS platform, including the creation, confirmation, and formatting of the request.

1. **Request Creation** (Fig 19)
  - Dr. Smith from Hospital A logs into the B-UMCS platform and navigates to the “Request Consultancy” tab.
  - Dr. Smith fills out the consultation request form, including the medical case details concerning the patient’s symptoms and the required specialist(s).
2. **Transaction Confirmation** (Fig 20)
  - The MetaMask interface prompts Dr. Smith to confirm the transaction details, including gas fees and the recipient’s public key (if encryption is required).
  - Upon confirmation, the request is broadcasted to the Ethereum blockchain. First, the request is added to the IPFS, and then the corresponding hash code is logged on the blockchain using an appropriate smart contract (Fig 6).
3. **Request Format** (Fig 21)
  - The request is formatted with a unique case number, message, sender name, sender public address, and any attached documents (EHR or Medical CR).

**Step 3: Requests Notification to Providers** (Figs 8 and 22): This step details how the healthcare providers are notified of the newly added consultation requests and how they can promptly access the necessary information.

1. **Notification of Consultation Request** (Fig 8)
  - **Fetch new requests from the blockchain**: Here, the hash code of the newly added request by Dr. Smith is fetched from the blockchain through smart contracts.
  - **Retrieve the request details from IPFS and visualize it to Hospitals**: The hash codes will be references to the details of Dr. Smith’s request saved on the IPFS that will be then returned and displayed on the concerned hospital homepage (e.g., Hospital B).
2. **Viewing Received Requests** (Fig 22)
  - Dr. Jones from Hospital B and Dr. Patel from Hospital D receive notifications of the consultation request.
  - Dr. Jones and Dr. Patel log into the platform and access the “Received Messages” tab to view the consultation request details.

**Step 4: Hospital Reply to the Request** (Figs 9 and 23): This step comes after the hospital has viewed the consultation request and decided to reply to it.

1. **Reply to the request at front-end**
  - Dr. Jones from Hospital B, after checking the case, decides to reply to Dr. Smith showing interest in offering a consultation service to this case. This is done through the B-UMCS reply interface at the front-end (Fig 23).
2. **Reply to the request at the back-end**
  - At the back-end, this reply is first uploaded to the IPFS, and then its hash code is logged on the blockchain through smart contracts (Fig 9).

**Step 5: Data Upload to IPFS** (Fig 24): This step explains how the patient’s EHR is uploaded to the IPFS and linked to the consultation request after Hospital B replies to Hospital A with its desire to share its expertise and consult the case.

1. **EHR Upload**
  - Dr. Smith uploads the patient’s EHR to the IPFS through the system interface (Fig 24), generating a unique cryptographic hash code.
2. **Hash Code Storage on Blockchain**
  - The hash code of the EHR is stored on the blockchain, linking the consultation request to the uploaded data (Fig 10). Only Hospital B can read this EHR (Fig 25).

**Step 6: Consultation and Update** (Figs 11, 29 and 30): This step describes the process of reviewing the EHR, providing recommendations, and updating the blockchain with new information.

1. **Review and Recommendations** (Fig 11)
  - Dr. Jones, after reviewing the case and the EHR, prepares his insights and recommendations.
2. **Updating the Blockchain and Consultation Report (CR) Notification** (Figs 29 and 30)
  - These recommendations are uploaded to IPFS as CR, generating a new hash value that is stored on the blockchain to ensure data integrity (Fig 29).
  - Dr. Smith is notified of the completed consultation and can access the CR, which is displayed through Dr. Smith Hospital A’s home pages (Fig 30).

This detailed scenario demonstrates the comprehensive functionality of the B-UMCS system, highlighting the secure and efficient handling of medical consultations using blockchain and IPFS technologies. Figs 2 to 34 provide visual clarity on the interaction among different components and the step-by-step execution of the system’s processes.

Therefore, to protect patient privacy, all EHRs are anonymized before being uploaded to IPFS. Access to these records is controlled through public key encryption, ensuring that only authorized individuals can view sensitive information. In scenarios where multiple specialists are consulted, the system uses RBAC to ensure that only concerned entities can access medical-related records. This ensures that patient data is only accessible to the relevant specialists, maintaining strict control over who can access specific types of medical information.

B-UMCS ensured scalability by utilizing the blockchain’s distribution nature and the IPFS structure. On the other hand, integrity is achieved through the blockchain architecture itself, the hash function in the IPFS, and the digital signatures.

Regarding the data retrieval speed and response time, the B-UMCS was prompt in this regard, assuming the medical consultants would handle the request once received, which might be different in real-life scenarios. A policy could be defined among the hospitals to put a due date/time for each request based on its priority which consequently might affect the calculation of the response time with respect to due time. This could be part of future work where tests are conducted in real-world scenarios involving hospitals and healthcare providers. These evaluations also include taking user feedback about their satisfaction with the system services.

## Conclusions and future work

Over the last decade, telehealth’s trajectory within the healthcare landscape had shifted from incremental adoption to becoming a critical component amidst the COVID-19 pandemic. This crisis spotlighted telehealth’s role in mitigating the challenges posed by a global shortage of medical professionals, underscoring the need for a robust, secure, and scalable system. Our research introduced a groundbreaking blockchain-based platform engineered to unify medical professionals across diverse geographic and institutional boundaries. Unlike existing solutions, our platform leveraged a unique integration of blockchain and the IPFS to ensure secure, transparent, and efficient healthcare consultancy services. This innovation not only facilitated the seamless exchange of EHRs and consultancy services but also ensured the utmost confidentiality, integrity, and availability of data, setting a new benchmark in telehealth service delivery. At the heart of our proposed system lies a decentralized framework, utilizing blockchain’s inherent security features to safeguard healthcare communications. The system architecture comprised five key elements: Hospitals, DApps, off-chain storage solutions, blockchain infrastructure, and smart contracts. Each component was meticulously designed to augment the system’s reliability, ensuring a seamless, secure healthcare service ecosystem. Through detailed real-world scenarios, our study demonstrated the platform’s potential to enhance healthcare delivery significantly, transcending traditional resource limitations. The implementation of such a system not only addressed immediate telehealth challenges but also paved the way for a future where healthcare accessibility is universal and unrestricted by geographical or institutional constraints.

While our system represented a significant leap forward, we acknowledge the challenges associated with implementing such a comprehensive platform at scale, including interoperability issues with existing healthcare systems and the need for widespread blockchain literacy among healthcare professionals. These challenges underscore the importance of continued research and collaboration to refine and enhance the system’s capabilities. Looking ahead to the future, we are committed to expanding the system’s functionality to include more stakeholders, such as patients, insurance providers, and government health agencies. Our goal is to develop features that further facilitate patient-centered care, improve healthcare outcomes, and ensure equitable access to medical expertise globally. Potential collaborations with technology developers and policymakers will be crucial in achieving these objectives, as will ongoing technological advancements that can enhance the platform’s performance and user experience. As part of our future work, we plan to integrate AI into the B-UMCS framework to further enhance its performance, particularly in areas such as data analysis, predictive analytics, and personalized healthcare recommendations.

The impact of our system on stakeholders is profound: patients gain access to a broader pool of medical expertise, healthcare providers can share knowledge and resources more efficiently, and institutions can deliver higher-quality care with enhanced data security. By addressing the critical need for a robust, scalable telehealth platform, our research contributes significantly to the sustainability and resilience of healthcare systems worldwide. In conclusion, we call upon the research community, healthcare policymakers, and technology developers to engage with our findings and contribute to the ongoing development of blockchain in healthcare. Together, we can overcome the current limitations and pave the way for a future where high-quality healthcare is universally accessible, supported by a blockchain-based consultancy service that redefines the paradigms of telehealth. Our work is but a step toward this ambitious vision, highlighting the transformative potential of blockchain technology in providing secure consultancy services and creating more connected and robust healthcare ecosystems.
